# Adult spiny mice (*Acomys*) exhibit endogenous cardiac recovery in response to myocardial infarction

**DOI:** 10.1038/s41536-021-00186-4

**Published:** 2021-11-17

**Authors:** Hsuan Peng, Kazuhiro Shindo, Renée R. Donahue, Erhe Gao, Brooke M. Ahern, Bryana M. Levitan, Himi Tripathi, David Powell, Ahmed Noor, Garrett A. Elmore, Jonathan Satin, Ashley W. Seifert, Ahmed Abdel-Latif

**Affiliations:** 1 Saha Cardiovascular Research Center, College of Medicine, University of Kentucky, Lexington, KY USA; 2 The Center for Translational Medicine, Lewis Katz School of Medicine, Temple University, Philadelphia, PA USA; 3 Department of Physiology, College of Medicine, University of Kentucky, Lexington, KY USA; 4 Gill Heart and Vascular Institute and Division of Cardiovascular Medicine, University of Kentucky, Lexington, KY USA; 5 Department of Biology, University of Kentucky, Lexington, KY USA; 6 The Lexington VA Medical Center, Lexington, KY USA; 7 Division of Cardiovascular Medicine, Department of Internal Medicine, University of Michigan, Ann Arbor, MI USA

**Keywords:** Cardiac regeneration, Experimental models of disease

## Abstract

Complex tissue regeneration is extremely rare among adult mammals. An exception, however, is the superior tissue healing of multiple organs in spiny mice (*Acomys*). While *Acomys* species exhibit the remarkable ability to heal complex tissue with minimal scarring, little is known about their cardiac structure and response to cardiac injury. In this study, we first examined baseline *Acomys* cardiac anatomy and function in comparison with commonly used inbred and outbred laboratory *Mus* strains (C57BL6 and CFW). While our results demonstrated comparable cardiac anatomy and function between *Acomys* and *Mus*, *Acomys* exhibited a higher percentage of cardiomyocytes displaying distinct characteristics. In response to myocardial infarction, all animals experienced a comparable level of initial cardiac damage. However, *Acomys* demonstrated superior ischemic tolerance and cytoprotection in response to injury as evidenced by cardiac functional stabilization, higher survival rate, and smaller scar size 50 days after injury compared to the inbred and outbred mouse strains. This phenomenon correlated with enhanced endothelial cell proliferation, increased angiogenesis, and medium vessel maturation in the peri-infarct and infarct regions. Overall, these findings demonstrate augmented myocardial preservation in spiny mice post-MI and establish *Acomys* as a new adult mammalian model for cardiac research.

## Introduction

Significant clinical advances for heart revascularization and next-generation medical therapies have improved the mortality rate after myocardial infarction (MI)^1^. The initial ischemic insult, even with timely revascularization, is followed by microvascular injury and infarct expansion leading to exacerbated damage^2^. Unfortunately, no therapies exist to limit myocardial damage from MI and the ensuing infarct expansion results in millions of recovering patients progressing to develop heart failure (HF). This is because the normal healing response to tissue injury in adult mammals is fibrotic repair; an effective short-term strategy, but one that leads to compromised tissue function in the long-term. Fibrotic repair is the default strategy in the adult heart where it is the leading cause of the clinical HF epidemic^3^. Multiple therapies developed for limiting infarct expansion or cardiac repair based on animal models have achieved only modest clinical success. A failure to translate basic research findings into successful clinical treatments can be traced, in part, to a lack of adult mammalian models possessing either ventricular regeneration or the ability for myocardial preservation.

In contrast to humans^4–6^, the ability to regenerate injured organs is widespread among vertebrates. Fishes, newts, and salamanders have extensive regenerative ability, and can functionally replace heart tissue after amputation or severe injury by expediting revascularization^7^ and by mobilizing a highly proliferative cardiomyocyte (CM) pool^8,9^. Specifically, zebrafish are a well-characterized model for adult cardiac regeneration with the documented ability to recover from a plethora of heart injuries including apical resection^10^, cryo-injury^11^, and coronary artery ligation. Neonatal mice are also capable of limiting the initial damage and repairing the myocardium after excision and ischemic injury during the first few days after birth^12^. In contrast, most adult mammals generally exhibit poor recovery capacity, especially as it pertains to recovering from heart damage. Spiny mice (*Acomys spp*.) are murid rodents found throughout Africa, the Middle East and Western Asia. These rodents exhibit a number of special traits;^13^ tantamount among them is the ability to regenerate skin, complex tissue^14–19^, and nephric tissue^20^. Recently, a large-scale survey of mammals suggested that spiny mice CMs exhibit a higher incidence of diploidy relative to laboratory mice^21^, while another study reported enhanced recovery after infarction injury^22^. Still, it remains poorly understood how spiny mice respond to heart injury and the mechanistic basis for their recovery remains unknown.

To rigorously examine how spiny mice respond to heart damage, we first detail *Acomys* heart structure and cardiac function in comparison to the most widely used inbred laboratory *Mus* model of cardiac injury (C57BL6). For comparative purposes we also used an outbred Swiss Webster strain from Charles River (CFW) to account for larger size and greater genetic diversity within our spiny mouse population. This cross-species characterization indicates similar cardiac structure and comparable baseline function among spiny mice and the examined mouse strains. We next conducted a comparative analysis of these animals in response to permanent coronary artery ligation (MI). In contrast to the mouse strains studied, *Acomys* showed significantly improved survival and enhanced myocardial preservation following ischemic injury. The ability of *Acomys* to recover from ischemic injury is coincident with greatly improved angiogenesis rarely seen in adult mammals. These findings in *Acomys* support a rapid angiogenic response into fibrotic tissue which limits scar spread and enhances ischemic tolerance in response to MI.

## Results

### Heart and coronary tree anatomy are similar among *Acomys* and *Mus* (C57BL6 and CFW)

**Fig. 1:**
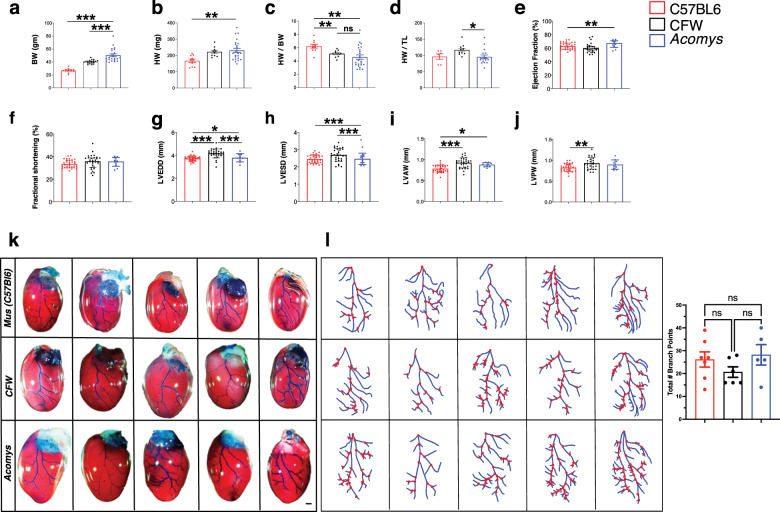
Characteristics of *Acomys* heart physiology and anatomy at baseline. **a**–**j** Measures of body weight (BW) and heart weight in C57BL6-*Mus* (*N* = 10), CFW-*Mus* (*N* = 19), and *Acomys* (*N* = 22). Analyses demonstrate comparable heart weight across species when normalized by body weight (BW) and tibia length (TL) (values are means ± S.E.M, *P < 0.05, **P < 0.01, and ***P < 0.001 by one-way ANOVA and Dunnett correction with *Acomys* as control). Ejection fraction (*F* = 4.1, *P < 0.05) (**e**), fractional shortening (*F* = 0.96, *P* > 0.05) (**f**), left ventricular end-diastolic diameter (LVEDD) (*F* = 14.8, **P < 0.01) (**g**), left ventricular end-systolic diameter (LVESD) (*F* = 4.6) (**h**), left ventricular anterior wall (LVAW) (*F* = 14.9, *P < 0.05) (**i**), left ventricular posterior wall (LVPW) (*F* = 6.4) (**j**) (*N* = 30 C57BL6-*Mus*, 10 CFW-*Mus* and 10 *Acomys*). Representative images showing comparable left anterior descending artery coronary anatomy across species using intra-aortic infusion of Batson’s 17 polymer mixture and corrosion casting (*N* = 5 each group, scale bar = 1 mm) (**k**). Quantification of coronary branching points of the arterial supply of the left ventricle across species. This analysis shows a comparable number of branching points across species with numerically higher (but not statistically significant) branching points in C57BL6-*Mus* and *Acomys* compared to CFW-*Mus* (**l**). Detailed statistical output for this figure can be found in the Supplementary Information.

To assess whether interpretable comparisons could be made across species, we first characterized hearts from *Acomys* and the most commonly used laboratory mouse (*Mus*) strain: C57BL6. In addition, we included an outbred Swiss Webster strain from Charles River (CFW) to control for increased size and genetic diversity in *Acomys*. To control for lifespan differences between species, we used sexually mature males, 6-month-old *Acomys*, and 8–12 week old *Mus* for these studies. First, we set out to determine whether baseline differences in cardiac gravimetrics exist between *Acomys* and *Mus* (C57BL6 and CFW). We assessed heart weight (HW) and normalized it to body size (body weight [BW] and tibia length [TL]) to account for overall size differences between species (Fig. 1a–d, and Supplementary Table 1). Although *Acomys* demonstrated a higher mean HW compared to C57BL6 (Fig. 1b), normalized to BW, *Acomys* was not significantly different compared to CFW and exhibited only a slightly lower HW/BW compared to C57BL6 (Fig. 1c). CFW mice demonstrated a slightly higher HW compared to *Acomys* and C57BL6 when normalized to TL (Fig. 1d). *Acomys* showed heavier wet lung weight compared to *Mus* (C57BL6 and CFW) (Supplementary Fig. 1a), but lower wet and dry lung weight after normalization by BW (Supplementary Fig. 1b, e). Importantly, dry lung weight before normalization by TL and dry and wet lung weight after normalization by TL were not significantly different across species (Supplementary Fig. 1d–f). The complete data is summarized in Supplementary Table 1.

Next, we compared cardiac functional parameters and observed comparable baseline cardiac function under physiological conditions across species. Global cardiac function as presented by left ventricular ejection fraction was statistically higher in *Acomys* compared to *Mus* (Fig. 1e), but fractional shortening was not significantly different across species (Fig. 1f). Measures of left ventricular cavity diameter were also comparable between species, with the exception of CFW-*Mus* showing slightly larger left ventricular end-diastolic diameter and thicker anterior wall compared to *Acomys* (Fig. 1g–j and Supplementary Table 1). We also assessed gross cardiac structure and coronary tree anatomy among *Acomys* and *Mus* (C57BL6 and CFW) and found that cardiac structure was not grossly different between the species upon visual comparison of whole hearts along the short- or long-axis (Supplementary Fig. 1g). Using corrosion casting and intra-aortic infusion of Batson’s 17 polymer mixture, we delineated the left coronary anatomy in all species. Relevant to permanent coronary ligation, all three demonstrated a similar course of the left anterior descending artery (LAD) supplying the anterior wall of the left ventricle (Fig. 1k). Coronary blood vessels adopt a hierarchical structure and vascular density leads to enhanced blood supply, angiogenesis, and ischemic resistance^23,24^. Thus, we analyzed the bifurcation points of the coronary tree as it supplies the anterior wall of the left ventricle. These analyses showed a similar density of branching points per major artery across species (Fig. 1l). Finally, we assessed capillary density at the mid left ventricular level between species and observed slightly higher capillary density in *Acomys* and CFW-*Mus* compared to C57BL6-*Mus* (Supplementary Fig. 2). While some of the baseline comparisons were statistically significant, these differences were not biologically relevant and did not correlate with post-injury response. These data demonstrated comparable cardiac anatomy and physiology between *Acomys* and the two *Mus* strains, C57BL6 and CFW.

### Adult *Acomys* exhibit a high percentage of cardiomyocytes possessing characteristics associated with immature *Mus* ventricular cardiomyocytes

**Fig. 2:**
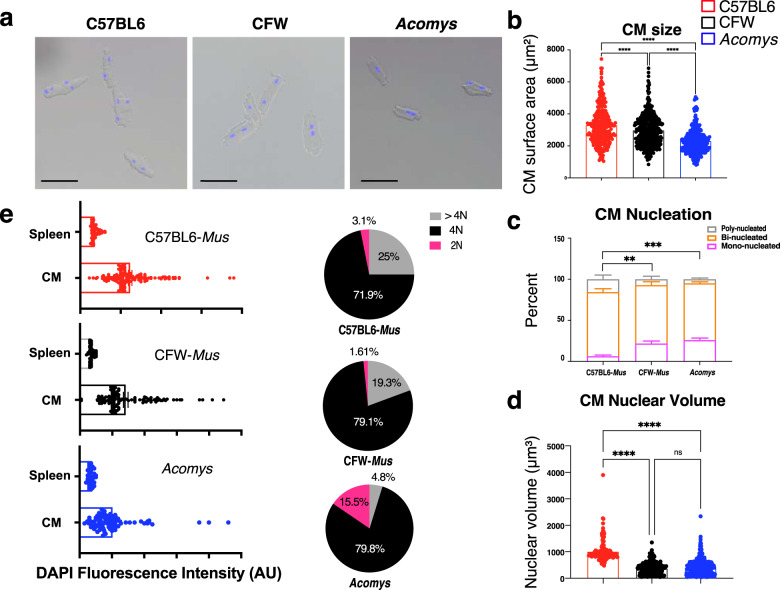
*Acomys* cardiomyocytes exhibit features associated with *Mus* immature cardiomyocytes. **a** Representative images of single-cell ventricular suspension stained with DAPI (blue), identifying small, mononuclear, and binucleated cardiomyocytes in *Acomys* and larger binucleated cardiomyocytes in *Mus*-C57BL6 with *Mus*-CFW cardiomyocytes being intermediate in size (scale bar = 50 μm) with mononuclear and binucleated cardiomyocytes. **b** Quantitative analyses of cell surface area of *Mus* and *Acomys* (*n* = 80–120 randomly selected CMs/animal and *N* = 4 each for *Mus*-C57BL6, *Mus*-CFW, and *Acomys*, ***P < 0.001 by one-way ANOVA; values are means ± S.E.M). **c** Percentage of mono-, bi- and poly-nuclear cardiomyocytes in C57BL6-, CFW-*Mus* and *Acomys* (*n* = 80–120 randomly selected CMs/animal, *N* = 4 mice for each group, values are means ± S.E.M, **P < 0.01, ***P < 0.001 by One-way ANOVA and Dunnett’s post-hoc analysis, compared to C57BL6-*Mus*). **d** Nuclear volume among isolated cardiomyocytes showing small nuclear volume in CFW-*Mus* and *Acomys* compared to C57BL6-*Mus* (*n* = 80–120 randomly selected CMs/animal, *N* = 4 mice per group, values are means ± S.E.M, ***P < 0.001 by One-way ANOVA and Dunnett’s post-hoc analysis, compared to C57BL6-*Mus*). **e** Violin plots, on left, demonstrating the distribution of DAPI fluorescence intensity in cardiomyocyte nuclei as assessed using Imaris on 3D (Z-stack) microscopy images. The right panel shows the percentage of CMs within each ploidy category from the total pool of CMs from each species (2N, 4N, and > 4N); *n* = 84–161 randomly selected CMs/species).

Ventricular CM phenotype varies across species and chronological age where it can influence the cardiac response to injury^12,21,25,26^. In mammals, a high percentage of mononuclear diploid CMs is observed in neonates and is indicative of an immature heart phenotype that is rarely seen in adult mammals^27,28^. To examine CM phenotype between species, we isolated ventricular CMs from *Mus* and *Acomys* and examined their size, number of nuclei/CM, nuclear characteristics, and ploidy (Fig. 2). Similar to a prior report^21^, *Acomys* CMs were significantly smaller than C57BL6 and were approximately four times more likely to be mononucleated (*Acomys*: 25.9 ± 5.1% vs. CFW-*Mus*: 21.7 ± 3% vs. C57BL6-*Mus*: 6.3 ± 2.8%, *P* < 0.001; Fig. 2a–c). Additionally, when we quantified the average nuclear volume across all CMs from *Acomys* and *Mus* we found that CM nuclei were smaller in *Acomys* (Fig. 2d)^29^. We then assessed the ploidy of ventricular CMs across species using 3-dimensional confocal microscopy^30^ and found a significantly higher percentage of diploid CMs in *Acomys* compared to *Mus* with a corresponding lower percentage of >4N CMs (Fig. 2e). Compared to C57BL6 CMs, CFW-*Mus* CMs were smaller with a higher percentage of CMs being mononuclear, a finding that is similar to CM data collected for the inbred SWR/J strain^25^.

**Fig. 3:**
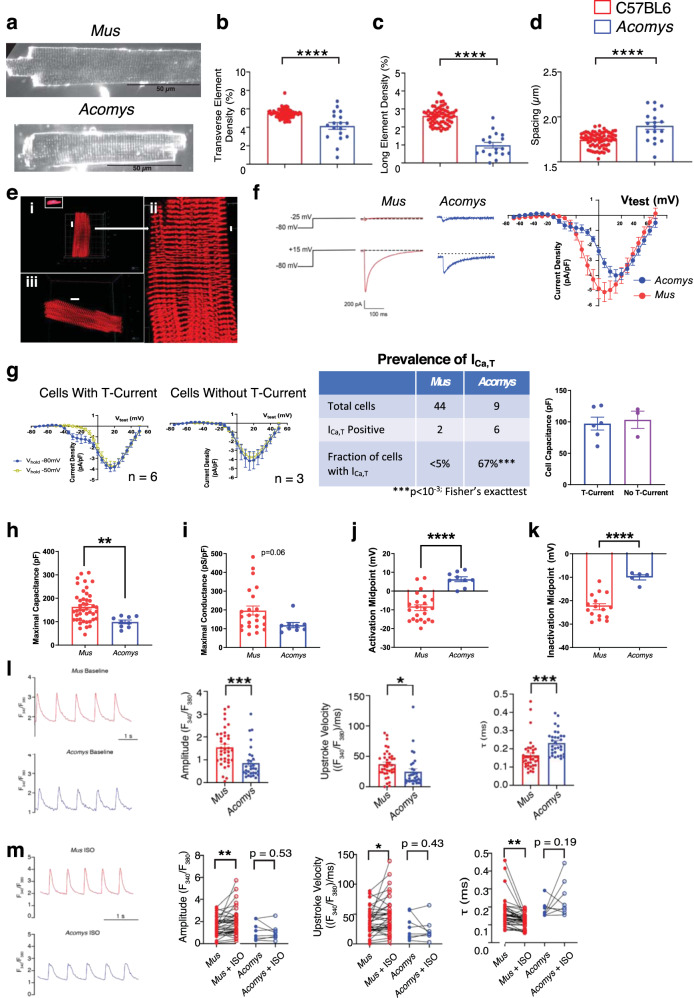
Physiological characteristics of *Acomys* and *Mus* cardiomyocytes. **a** Representative images of isolated cardiomyocytes from C57BL6-*Mus* and *Acomys* stained with di-8-ANEPPS to visualize T-tubules. T-tubule analysis shows significantly reduced T-tubule density in transverse (**b**) and axial directions (**c**) along with wider T-tubule spacing (**d**) in *Acomys* compared to *Mus* (C57BL6; *t* = 6.2, 12, and 4.4; *F* = 39, 141, and 19, for **b**–**d**, respectively; *Mus* *N* = 5, *n* = 67; *Acomys* *N* = 3, *n* = 23. **e** *Acomys* ventricular cardiomyocytes show organized z-discs. (i) Mononucleated CM α-actinin reveals highly organized z-disc structure (scale bar = 10 μm). (ii) expanded view of mononucleated CM in **i** (scale bar = 2 μm). (iii) Representative binucleated CM (scale bars = 10 μm). **f** Current traces (left) current-voltage relationship (right) elicited by V_test_ −25 mV (left, upper) and V_test_ +15 mV (left, lower) from V_hold_ −80 mV. **g** (Left) Current-voltage curves from V_hold_ −80 mV and −50 mV superimposed for *Acomys* CM exhibiting I_Ca,T_ (A) or no I_Ca,T_. **g** (Left center) I_Ca,T_ expression was heterogeneous but more *Acomys* ventricular CMs showed I_Ca,T_ compared to rare occurrences in *Mus*-C57BL6 (**g**, right center). **g** (Right) Cell capacitance was not different for *Acomys* CM with or without I_Ca,T_. **h** Cell capacitance was greater in *Mus*-C57BL6 compared to *Acomys* (*t* = 2.88, *F* = 7.9, **P < 0.01). **i** Maximal conductance density trended greater in *Mus*-C57BL6 compared to *Acomys* (*t* = 2.0, *F* = 7.7, *P* = 0.06). **j**–**k** Voltage-dependent activation and inactivation of I_Ca,L_ was significantly shifted positive for *Acomys* compared to *Mus*-C57BL6 (*t* = 5.8 and 5.2, *F* = 3.7 and 4.5, for **i**, **j**, respectively, ****P < 0.001). **l** Representative calcium transients from isolated ventricular cardiomyocytes loaded with fura2-AM, *Mus*-C57BL6 (top, red) and *Acomys* (bottom, blue) paced at 1 Hz. Scale bar: 2 s. **l** (Left) Amplitude of the transients (*t* = 3.7, *F* = 1.4, ***P = 0.0004). **l** (Middle) Velocity at which calcium enters the cytosol (upstroke of the transient: *t* = 2.1, *F* = 1.5, *P = 0.04). **l** (Right) (τ) Calcium transient decay (*t* = 3.6, *F* = 1.5, ***P = 0.0005). **m** Representative calcium transients treated with 100 nM isoproterenol (ISO), *Mus*-C57BL6 (top, red) and *Acomys* (bottom, blue), paced at 1 Hz. Scale bar: 2 s. (Left) Before and after ISO Amplitude of the transients (*t* = 2.8 for Mus, *P* = 0.53, *t* = 0.7 for *Acomys*, **P = 0.007). (Middle) Before and after ISO Velocity at which calcium enters the cytosol (*t* = 2.5, *P* = 0.02). (Right) Calcium transient decay (*t* = 3.4 for *Mus*, **P = 0.002; *N* = 7 animals, *n* = 37 cells for C57BL6-*Mus*; and *N* = 4 animals, *n* = 31 cells for *Acomys*).

The relatively small cell size and higher mononucleation we observed in *Acomys* CMs suggested a higher percentage of CMs exhibiting characteristics observed in immature *Mus* CMs. T-tubule organization is highly regular in mature mouse CMs^31^. To assess T-tubule organization we stained CMs with di-8-ANEPPS (Fig. 3a) and evaluated transverse and axial density along with T-tubule spacing. *Acomys* showed significantly reduced transverse and long element densities (Fig. 3b, c) with increased resting T-tubule spacing (Fig. 3d). In contrast to less regular T-tubule organization, *Acomys* showed highly registered z-discs (Fig. 3e and Supplementary Movie 1). To further explore the physiological implications of these findings, we conducted electrophysiological studies on isolated ventricular CMs from C57BL6-*Mus* and *Acomys*. In mammalian hearts, the expression of low-voltage activated (T-type calcium channels) is an index of the fetal gene program in the ventricle;^32^ therefore, we recorded voltage-dependent I_Ca_ from *Acomys* and *Mus* ventricular myocytes. From a V_hold_ −80 mV, a V_test_ step to −25 mV elicited little if any discernible current in *Mus* (C57BL6) whereas, at V_test_ −25 mV, *Acomys* CMs exhibited a transient ‘T-type’ calcium current (I_Ca,T_) (Fig. 3f, upper panel). Longer-lasting calcium current (I_Ca,L_) was prominent with larger depolarizations in *Mus* (C57BL6) and *Acomys* (Fig. 3f, lower panel). The current-voltage relationship showed a prominent I_Ca,T_ component with a peak current between V_test_ −20 and −25 mV in *Acomys*, but not in *Mus* (C57BL6) (Fig. 3f).

To further dissect I_Ca,T_ from I_Ca,L_, we performed current-voltage curve protocols from V_hold_ −50 mV to voltage-inactivate the I_Ca,T_ component (Fig. 3g). Current-voltage relationships for I_Ca,T_-expressing *Acomys* cells showed the prominent I_Ca,T_ appearing as a low-voltage activated current for V_hold_ −80 mV but not from V_hold_ –50 mV (Fig. 3g, left). 33% of *Acomys* ventricular CMs showed no I_Ca,T_ (Fig. 3g, center) but there was no observed correlation of cell size to presence of I_Ca,T_ (Fig. 3g, right). Cell capacitance was significantly lower in *Acomys* compared to *Mus* (C57BL6) (Fig. 3h), consistent with our morphometric analysis (Fig. 2b). Additionally, maximal conductance density trended greater in *Mus* (C57BL6) than *Acomys* (Fig. 3i). Voltage-dependent activation and inactivation of I_Ca,L_ was significantly shifted positive for *Acomys* compared to *Mus* (C57BL6) (Fig. 3j, k).

We next explored cytosolic calcium handling. Cytosolic Ca^2+^ transients (CaT) had a larger amplitude (Fig. 3l, left), faster upstroke (Fig. 3L, middle), and more rapid decay (Fig. 3l, right) in *Mus* (C57BL6) compared to that observed in *Acomys*. As with CM morphometrics (Fig. 2b–d) and the prevalent expression of a T-type calcium current (Fig. 3f, g) the smaller, slower CaT segregated with a less mature CM phenotype. Immature ventricular CMs also tend to show reduced β-adrenergic receptor (β-AR) acute responsiveness^33^. Therefore, we tested the effect of isoproterenol challenge (ISO). To assess acute ISO responsiveness, we compared within-cell before and after ISO. *Mus* (C57BL6) showed increased amplitude and more rapid kinetics (Fig. 3m). By contrast, *Acomys* CaT amplitude and upstroke velocity were not significantly different (*P* > 0.05). Ca^2+^ re-uptake was accelerated by ISO in *Mus* but not in *Acomys* (Fig. 3m, right). Taken together, these data are consistent with an *Acomys* CM phenotype distinct from that of C57BL6-*Mus*. Moreover, these data demonstrate that a high percentage of *Acomys* CMs exhibit characteristics usually associated with *Mus* neonatal CMs.

### *Acomys* hearts demonstrate enhanced cardiac preservation after ischemic injury

**Fig. 4:**
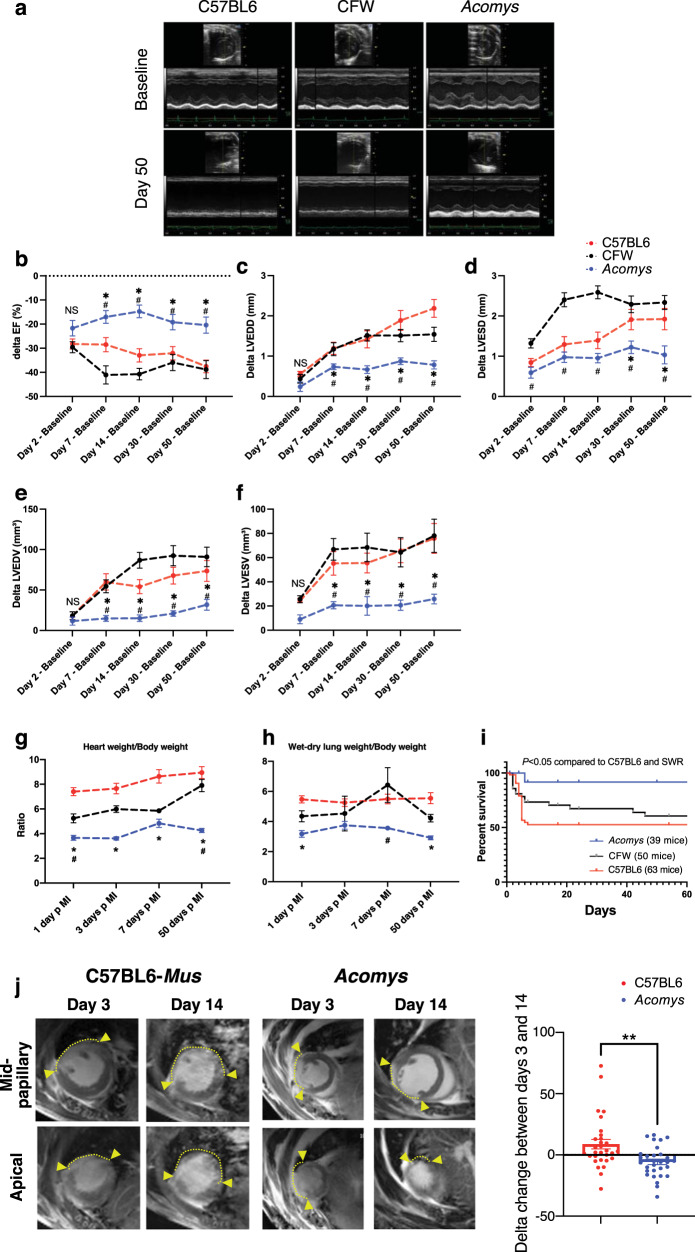
Myocardial ischemic injury response in *Acomys* and *Mus*. **a** Representative M-mode echocardiography images of *Mus*-C57BL6 (left), *Mus*-CFW (middle), and *Acomys* (right) at baseline (upper images) and day 50 after MI (lower images). Delta (Δ) ejection fraction (EF) (**b**), Δ left ventricular end-diastolic diameter (LVEDD) (**c**), Δ left ventricular end-systolic diameter (LVESD) (**d**), Δ left ventricular end-diastolic volume (LVEDV) (**e**), and Δ left ventricular end-systolic volume (LVESV) (**f**) after injury, calculated as difference between each time point and baseline value in the same animal (*N* = 16–45 *Mus*-C57BL6, 10–20 *Mus*-CFW and 8–11 *Acomys*) (Values are means ± S.E.M, *P < 0.05 by Mixed-effects ANOVA, compared to *Mus-C57BL6 or ^#^*Mus*-CFW). **g** Quantitation of heart weight (HW) normalized by body weight (BW) for *Mus*-C57BL6 (*N* = 2–26 animals), *Mus*-CFW (*N* = 3–11 animals) and *Acomys* (*N* = 2–13 animals) at baseline and various timepoints after MI suggesting maintenance of HW/BW ratio in *Acomys* and progressive increase in *Mus* (Values are means ± S.E.M, *P* < 0.05 by two-way independent ANOVA, compared to *Mus-C57BL6 or ^#^*Mus*-CFW). **h** Quantitation of wet-dry lung weight normalized by body weight of *Mus*-C57BL6 (2–26 animals), *Mus*-CFW (3–11 animals) and *Acomys* (1–13 animals) at various timepoints after MI suggesting maintenance of wet-dry lung weight/body weight ratio in *Acomys* and progressive increase in *Mus* (Values are means ± S.E.M, *P < 0.05 by two-way independent ANOVA, compared to *Mus-C57BL6 or ^#^*Mus*-CFW). **i** Kaplan–Meier analysis depicting mortality after MI and showing significantly lower mortality in *Acomys* compared with *Mus* (Gehan–Bareslow–Willcoxon test, *P* < 0.05). **j** Representative cardiac magnetic resonance (CMR) images showing comparably medium-sized infarct area in both species at 3 days post MI (arrows show the boundaries of late gadolinium enhancement/injury). The progression of infarct area was significantly slower in *Acomys* compared to *Mus* at 14 days after MI (*N* = 9–10 animals per group, *t* = 3.28, *P < 0.05 by paired *T*-test).

*Acomys* can regenerate complex tissue wounds, but the response to myocardial ischemic (MI) injury has not been extensively explored. Thus, we sought to compare the cardiac injury response between *Acomys* and the two strains of *Mus* using the permanent LAD-ligation model of MI^34–36^. After injury, all groups showed a comparable drop in cardiac function, reflecting similar injury response as assessed by serial echocardiography (Fig. 4, Supplementary Fig. 3 and Supplementary Table 1). Indeed, there was no significant difference in most cardiac functional parameters 48 h after MI (Fig. 4b–f, *P* > 0.05). When we assessed the extent of cardiac tissue injury in a subset of animals 3 days after MI using Masson’s Trichrome and cardiac troponin T staining, we observed a comparable degree of CM loss and tissue injury between *Acomys* and the C57-*Mus* mice (Supplementary Fig. 3a, b). Furthermore, our cardiac MRI imaging showed comparable injury and involvement of the distal anterior wall and apex 3 days after MI between species (Supplementary Movies 2–5) as demonstrated by a similar area of late gadolinium enhancement (Supplementary Figs. 4 and 5). Our long-term echocardiography analyses showed stabilization in cardiac function and remodeling parameters in *Acomys* compared to the progressive deterioration seen in *Mus* strains (C57BL6 and CFW) up to 50 days after injury (Fig. 4b–f). In these analyses, we utilized the delta change from baseline to account for any differences in baseline function between species which allowed us to isolate the analyses to the changes occurring after MI. We observed consistent changes in left ventricular global ejection fraction, as well as measures of left ventricular adverse remodeling such as end-systolic and end-diastolic diameters and volumes (Fig. 4a–f and Supplementary Fig. 3c–g). The enhanced cardiac preservation and reduced adverse cardiac remodeling in *Acomys* was reflected by lower heart/BW ratio throughout the follow-up period extending to 50 days after MI (Fig. 4g), as well as the aforementioned echocardiographic evidence of smaller ventricular diameters. We also observed significantly lower wet-dry lung weight in *Acomys* compared to *Mus* which is a measure of pulmonary edema and HF (Fig. 4h). Importantly, enhanced myocardial preservation in *Acomys* compared to *Mus* was associated with a significant survival advantage (Fig. 4i). As seen in prior reports, the majority of mortality across all species was observed in the first week after MI. While we observed cardiac rupture in 17 C57BL6 and 14 CFW mice, we observed none in *Acomys* as visualized in post-mortem necropsies.

**Fig. 5:**
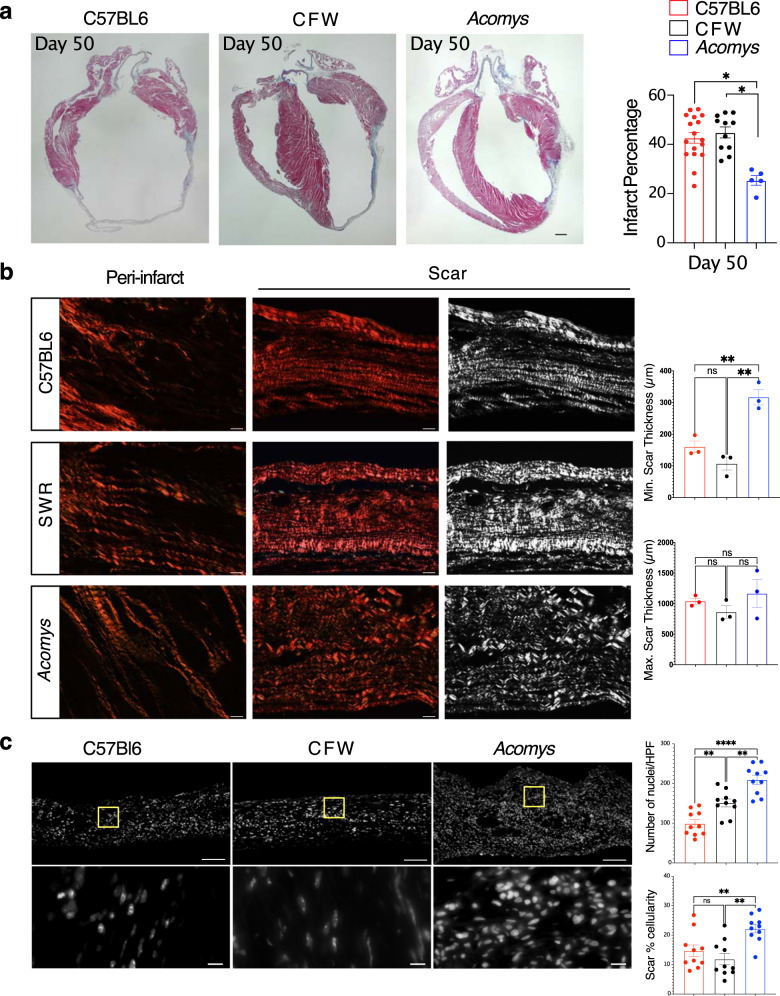
*Acomys* exhibit cardiac tissue preservation in response to myocardial infarction. **a** Representative images of Masson’s trichrome stained long-axis left ventricular cavity sections of *Mus* (C57BL6 and CFW) and *Acomys* 50 days post-MI (scale bar = 500 μm). Quantification of the infarct size, corresponding to the ratio between infarcted length and left ventricular length showing significantly smaller scar in *Acomys* compared to *Mus* strains (*N* = 17 *Mus*-C57BL6, 11 *Mus*-CFW, and 5 *Acomys*, values are means ± S.E.M, *P < 0.05 by one-way ANOVA and Tukey’s multiple comparison test). **b** Representative images of picrosirius red stained scar center 50 days after MI across species showing reduced alignment of collagen fibers in *Acomys* compared to *Mus* species. Both *Mus* strains show highly compressed, parallel collagen fibers, while *Acomys* fibers show more porosity between fibers and a wavy organization. Quantification of minimum and maximum scar thickness showing that *Acomys* has on average a comparatively thicker scar compared to *Mus* strains (*N* = 3 animal/group, values are means ± S.E.M, **P < 0.01 by one-way ANOVA and Tukey’s multiple comparison test). There was no statistically significant difference in maximum scar thickness. **c** DAPI images were obtained in the center of the scar and demonstrate higher cellularity in *Acomys* compared to *Mus* strains (scale bar = 100 μm in upper panel and 10 μm in lower panel, yellow boxes indicating insets). Quantitative analysis showed higher density of nuclei and scar percentage cellularity in *Acomys* vs. *Mus* strains (*N* = 3 animal/group, values are means ± S.E.M, **P < 0.01 by one-way ANOVA and Tukey’s multiple comparison test).

**Fig. 6:**
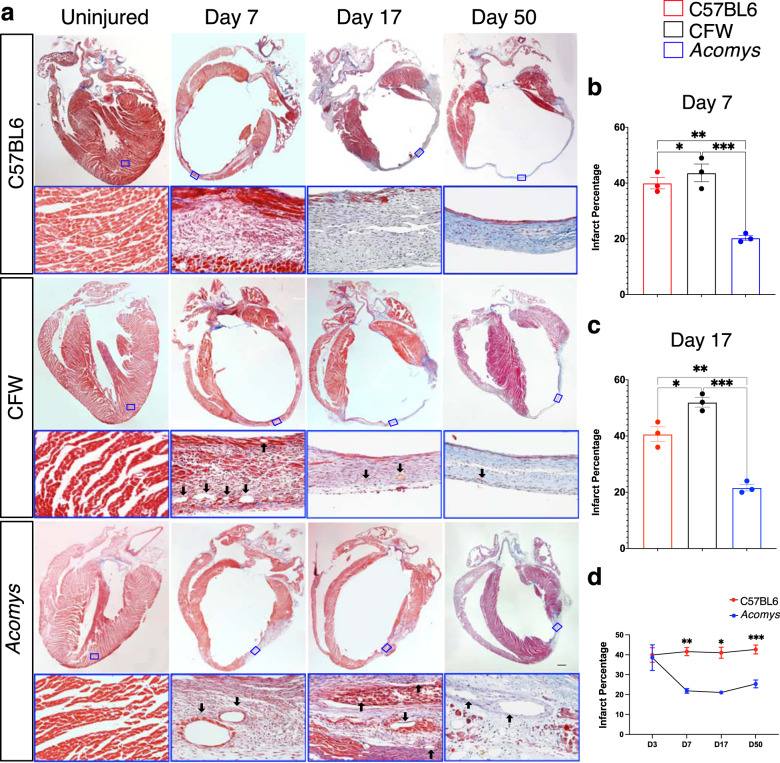
Cardiac scar tissue exhibits limited expansion in *Acomys*. **a** Representative images of Masson’s trichrome stained long-axis left ventricular cavity sections of *Mus* (C57BL6 and CFW) and *Acomys* in uninjured and MI animals at 7-, 17- and 50-days post-MI showing the progression of scar across species (scale bar = 500 μm). The lower panels represent the boxed areas and show the composition of the scar at each stage. **b**, **c** Quantification of the infarct size, corresponding to the ratio between infarcted length and left ventricular length showing significantly smaller scar in *Acomys* compared to *Mus* strains (*N* = 17 *Mus*-C57BL6, 11 *Mus*-CFW, and 5 *Acomys*, values are means ± S.E.M, *P < 0.05 by one-way ANOVA and Tukey’s multiple comparison test). **d** Scar progression in *Acomys* and *Mus*-C57BL6 showing dynamic reduction in infarct size in *Acomys* in contrast to the larger static scar in *Mus*-C57BL6 (*P < 0.05, **P < 0.01, and ***P < 0.001 as analyzed using 2-way independent ANOVA).

CMR studies demonstrated reduced scar progression between day 3 (D3) and D14 in *Acomys* compared to the large expanding infarct seen in *Mus* (Fig. 4j). Our long-term follow-up studies reflected significantly smaller infarct size in *Acomys* compared to *Mus* (C57BL6 and CFW) when assessed using Masson’s Trichrome staining 50 days after MI (Fig. 5a). We also saw similar results in our studies quantifying fibrosis using Picrosirius red staining 50 days after MI (Supplementary Fig. 3h). Using these Picrosirius stained sections to further explore the fibrous tissue alignment of the thicker scar in *Acomys*, we found that while the fiber alignment in the *Mus* strains was typical of a scar with highly parallel and compressed collagen bundles, fiber organization in the center of the *Acomys* scar was more porous with less compression (Fig. 5b). Furthermore, the resultant scar in *Acomys* was thicker across the entire scar which had a significantly higher cellular density (Fig. 5c), a finding that could potentially explain the lower rupture and mortality rates (Fig. 4i). Moreover, our serial scar analysis at D3 (Supplementary Fig 3a), 7, 17, and 50 (Fig. 6a–d) after MI demonstrated a unique evolution in infarct size between *Mus* and *Acomys* which corroborated the CMR studies (Fig. 4j and Supplementary Figs. 4 and 5). Taken together, our results demonstrate that *Acomys* exhibit ischemic tolerance and enhanced myocardial preservation after ischemic injury compared to the inbred C57BL6- and outbred CFW-*Mus* strains, respectively.

### *Acomys* exhibit rapid and enhanced vascularization of the infarct region after ischemic injury

**Fig. 7:**
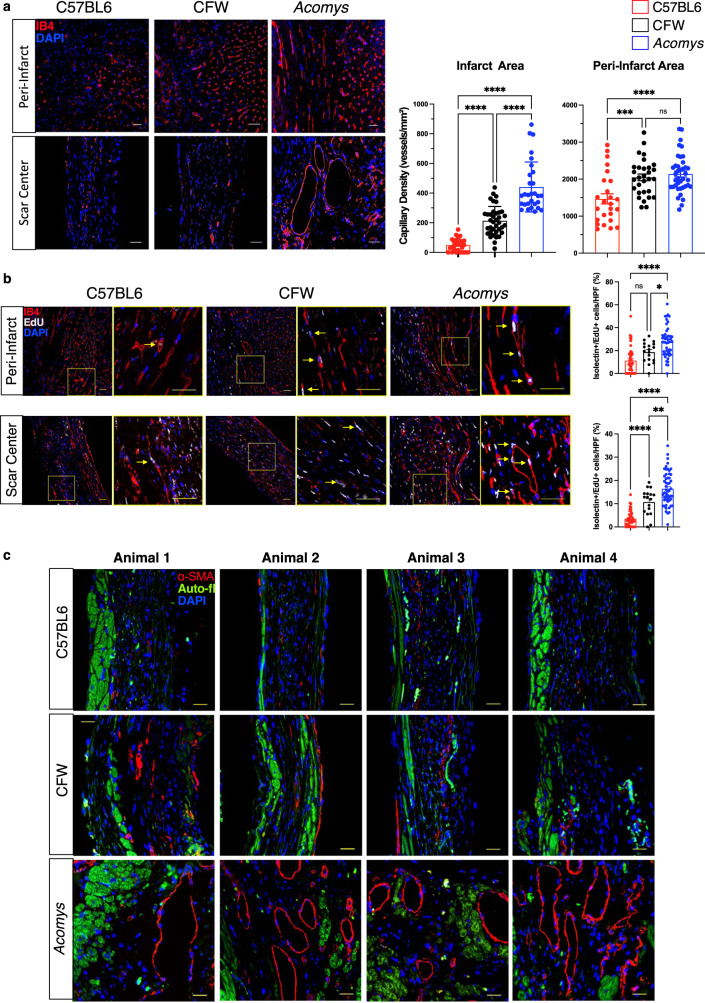
Enhanced angiogenesis and vessel maturation in the infarct region following MI in *Acomys* compared to *Mus* strains. **a** Representative images of CD31 staining (red) defining endothelial cells in the peri-infarct region and the center of the scar at the left ventricular cavity level of *Mus* (C57BL6 and CFW) and *Acomys* 50 days post-MI (scale bar = 25 μm). Quantification of capillary density showing significantly higher capillary density in *Acomys* compared to *Mus*-C57BL6 in the peri-infarct region and to both *Mus* strains in the center of the scar (*N* = 4 *Mus*-C57BL6, 4 *Mus*-CFW, and 5 *Acomys*, values are means ± S.E.M, ***P < 0.001 and ****P < 0.0001 by one-way ANOVA and Tukey’s multiple comparison test). **b** Representative images of CD31 (green, pseudocolored) and EdU staining (magenta) marking proliferating endothelial cells in the peri-infarct region and the center of the scar in *Mus* (C57BL6, left, and CFW, center) and *Acomys* (right) 17 days post-MI (scale bar = 25 μm). Insets show higher magnification of vascular structures. Quantification of percentage of CD31+ cells expressing EdU, showing significantly higher endothelial cell proliferation in *Acomys* compared to the two *Mus* strains in both peri-infarct and scar center areas (*N* = 4 *Mus*-C57BL6, 4 *Mus*-CFW, and 5 *Acomys*, values are means ± S.E.M, ****P < 0.0001 by one-way ANOVA and Tukey’s multiple comparison test). **c** Representative images of smooth muscle actin staining (red) in the scar center region showing higher prevalence of medium-sized blood vessels in *Acomys* compared to *Mus* strains. Autofluorescence (green) was used to identify the infarct boundary (images shown from 4 animals/group, scale bar = 25 μm).

Blood vessel formation following ischemic injury is essential for functional stabilization. Following ischemic injury, vascular damage ensues in the area surrounding the original infarct region and leads to infarct expansion, development of adverse cardiac remodeling and HF^2,37–39^. Several studies have shown successful reduction of infarct expansion through effective angiogenesis and vascular maturation^40,41^. We assessed the vascular density in the peri-infarct region in *Acomys* and *Mus* strains and observed an increased density of capillaries (as assessed by CD31 staining) in *Acomys* compared to *Mus* (Fig. 7a). As commonly observed in adult mammals following MI, both *Mus* strains exhibited reduced vascular density in the scar center; by contrast, *Acomys* showed enhanced vascular density extending into the scar center (Fig. 7a). Additionally, enhanced angiogenesis was linked to more proliferating endothelial cells (isolectin CD31+/EdU+) in the peri-infarct region and scar center in *Acomys* compared to both *Mus* strains (Fig. 7b). We confirmed these findings using isolectin B4 staining for endothelial cells. These analyses corroborated our CD31 results demonstrating higher capillary density in the center of the scar and the higher proliferative nature of endothelial cells (Supplementary Fig. 6). In support of enhanced angiogenesis, we observed a significantly higher density of mature blood vessels (α-SMA+) in the peri-infarct region in *Acomys* while the presence of these mature vessels was negligible in *Mus* strains (Fig. 7c). Taken together, in response to MI our data support rapid and enhanced angiogenesis followed by vascular maturation in *Acomys* compared to *Mus*. This can explain, at least in part, the ischemic tolerance, reduced damage area and the thicker, cellularly dense scar in *Acomys* compared to *Mus*.

## Discussion

Spiny mice (*Acomys*) represent a unique mammalian model to explore endogenous cardiac repair consistent with their enhanced regenerative ability for a number of tissues and organs. In this study, we established the anatomical, functional, and cellular characteristics pre- and post-MI in *Acomys* compared to the inbred laboratory mouse strain C57BL6 and the outbred CFW strain. Our results demonstrated comparable cardiac structure, coronary anatomy, and functional parameters between the species allowing us to appropriately use these animal models for comparative heart injury studies. Interestingly, when comparing CMs across species we found that adult *Acomys* possessed a distinct CM phenotype that included features usually observed in immature or fetal *Mus* CMs such as small size, mononucleation, T-type calcium current, reduced t-tubule density and organization. After ischemic injury, *Acomys* exhibited enhanced ischemic tolerance and significant myocardial preservation, resulting in reduced adverse cardiac remodeling, smaller scar size and, importantly, better survival. Overall, our data support that *Acomys* show resistance to the type of cardiac damage that compromises other adult mammals, including humans, and supports future cardiac studies using this model to explore the mechanisms underlying their enhanced angiogenic response to myocardial infarct (MI).

Similar to other highly regenerative vertebrates, *Acomys* have emerged as a bona fide mammalian regeneration model for their ability to naturally regrow complex tissues following full-thickness skin injury^14–19^ and excision of musculoskeletal tissue from the ear pinna^16,18,42^. In each type of injury, adult *Acomys* were capable of restoring functional tissues, a phenotype that has not been observed in other adult rodents. Our gross anatomical studies established a similar coronary anatomy across species^43^ and we found that *Acomys* hearts were similar structurally and anatomically to *Mus*. Importantly, in vivo cardiac functional parameters were comparable between the three groups. These findings provided the appropriate foundation for the current study and lay the groundwork for follow-up studies that can inform new therapeutic strategies for ischemic heart disease.

Increased mortality and incidence of HF following MI remain prevalent in modern medicine. While advanced revascularization therapies and mechanical support have slightly improved survival in patients with MI, millions develop progressive HF every year. Remarkably, *Acomys* exhibited significantly lower mortality after MI compared to both *Mus* strains and this reduction in mortality was related to a reduced incidence of cardiac rupture among *Acomys*. In fact, our histological assessment combined with cardiac MRI studies indicated significant differences in infarct evolution between species. While *Mus* demonstrated large infarct size, associated with thinning of the anterior wall, *Acomys* showed reduction in damage area despite starting with a comparable area of injury. These differences in infarct progression led to favorable cardiac remodeling and functional preservation demonstrated in our echocardiography studies.

This unique phenomenon was associated with a rapid angiogenic response that eventually led to superior ischemic tolerance. *Acomys* scars were thicker, more cellularized and had higher capillary and mature vessel density compared to *Mus*. This could at least partly explain the dramatically lower cardiac rupture rate and enhanced survival we observed in *Acomys* including the stabilization of their cardiac function during long-term follow-up. A recent study found evidence that *Acomys* recovered many smaller coronary blood vessels 28 days after an LAD injury, evidence which supports our results and the utility of *Acomys* as a unique model of cardiac recovery^22^. While the data presented in this paper does not indicate cardiac regeneration to pre-injury levels, the dramatic ischemic tolerance and myocardial preservation noted in *Acomys* was associated with an enhanced angiogenic response. In fact, myocardial injury after MI is related to tissue edema and microvascular obstruction and our CMR and IHC assessments show significant reduction in damage area between D3 and 7 in *Acomys*. These dynamic changes could be related to rapid resolution of tissue edema which is closely linked to enhanced angiogenesis. Indeed, preclinical and human MI studies have linked enhanced angiogenesis and vessel maturation with lower rates of infarct expansion^41,44^. While our data do not exclude the possibility that *Acomys* vascular cells are more resistant to ischemia, this presumed mechanism is less likely to explain the increased vascular density in the infarct region given the observed superior endothelial cell proliferation and vessel maturation noted in *Acomys* in our studies.

The mammalian heart loses its reparative capability after the first week of life where heart damage results in permanent loss of myocardium combined with fibrotic scarring and adverse remodeling^12^. The transition to fibrotic repair is closely linked with the loss of CM proliferation as CMs enter cell cycle arrest and become mononuclear polyploid or multinucleated^12,45^. However, in other vertebrate species where CMs maintain the lifelong ability to proliferate (e.g., zebrafish and newts), cardiac regeneration is still possible^45,46^. One of the hallmarks of regenerative hearts is the relatively high frequency of mononucleated diploid CMs^45–48^. This evidence suggests that higher proportions of mononuclear diploid CMs are associated with higher regenerative potential following ischemic injury^25^. Interestingly, our results showed that outbred CFW-*Mus* possessed an intermediate CM phenotype between *Acomys* and C57BL6 with respect to CM size and nuclei number. Despite possessing this CM phenotype, outbred CFW had nearly the same mortality as C57BL6 suggesting that rapid angiogenesis may be a key component to facilitate the protective features conferred by having a larger population of smaller, mononuclear CMs. Recent studies across a large number of mouse strains suggest additional CM characteristics, including their metabolic profile, might act as key elements in cardiac recovery after ischemic injury^26^. These characteristics were not fully explored in our study and thus may have contributed to the limited recovery seen in our CFW strain. On the other hand, cardiac tissue requires vascularization for supporting the high metabolic activity of CMs^37^, and our current findings support rapid angiogenic invasion into new fibrotic tissue as a mechanism to resist cardiac damage. Future studies are necessary to explore CM behavior across species and how this may relate to enhanced angiogenesis.

There are two types of Ca^2+^ channels in CMs, L-type, and T-type. L-type Ca^2+^ channels are highly expressed in the adult heart and are important therapeutic targets for the management of various cardiovascular diseases. In contrast, T-type Ca^2+^ channels are rarely found in adults and are present in fetal and early postnatal mice^49^. In our cell electrophysiology studies, adult *Acomys* exhibited a higher percentage of T-type Ca^2+^ channels. This feature is consistent with a higher prevalence of phenotypically ‘young’ CMs in *Acomys* compared to *Mus* (C57BL6). Furthermore, ventricular CMs in adult *Acomys* showed reduced Ca^2+^ transient amplitude, slower kinetics, and attenuated β-AR responsiveness. While this unique phenomenon in *Acomys* was associated with superior myocardial preservation after ischemic injury, because we did not examine Ca^2+^ channels in outbred CFW mice, we do not know if this feature is associated with the prevalence of small, diploid, mononucleated CMs. However, it is interesting to note that although our CM characterization studies showed that outbred CFW mice possessed a higher prevalence of small, diploid, mononucleated CMs compared to those from the C57BL6 strain, this feature alone did not confer the type of enhanced myocardial preservation response observed in previous work using inbred SWR/J mice^25^. This would suggest a synergy between CM phenotype and enhanced angiogenesis with respect to cardiac preservation following MI. Future studies are necessary to examine the proliferative potential of adult CMs in *Acomys* and *Mus* as well as their potential to adequately respond to myocardial injury.

In conclusion, our study presents *Acomys* as a unique model for cardiac research with comparable cardiac structure, function, and anatomy to C57BL6- and CFW-*Mus*. *Acomys* exhibited a distinct CM phenotype and angiogenic response resulting in enhanced recovery and ischemic tolerance after ischemic injury. It is important to note that while we do not see clear evidence of cardiac regeneration, the enhanced myocardial preservation seen in *Acomys* could uncover important therapeutic targets for millions of patients who develop ischemic cardiomyopathy after MI. Future studies will focus on the mechanism and potential for these phenotypic differences to foster myocardial preservation and repair instead of scarring and HF in models of cardiac disease.

## Methods

### Animal care

Male *Acomys cahirinus* (6–8 months old, sexually mature animals) were obtained from our in-house breeding colony. Male *Mus musculus* (8–12 weeks, sexually mature animals) C57BL6/J (strain #000664) were obtained from the Jackson Laboratory, Bar Harbor, ME, and outbred Swiss Webster (CFW) were obtained from Charles River (strain # 024). Spiny mice were housed in their own building where they were maintained on an average 12:12 h L:D cycle with exposure to natural light and maintained on a diet of 14% mouse chow (Teklad Global 2014, Harlan Laboratories) along with black sunflower seeds. *Mus* strains were maintained by the University of Kentucky, Division of Laboratory Animal Resources and were exposed to a strict 12:12 h L:D cycle and maintained on an 18% mouse chow (Tekland Global 2918, Harlan Laboratories, Indianapolis, IN) diet. All animal procedures were approved by the University of Kentucky Institutional Animal Care and Use Committee (IACUC #: 2019-3254, 2013-1155, 2021-3739, and 2011-0889).

### Coronary anatomy

Coronary anatomy was visualized using corrosion casting technique. Mice received an i.p. injection of heparin (100 units in 300 μl sterile PBS) to prevent coagulation. Ten minutes later, under isoflurane anesthesia, hearts were excised and cannulated with a blunt tip 23 g needle and clamp. 10 ml of 1× PBS was perfused via ascending aorta, followed by 1.5 ml Batson’s 17 polymer mixture (2.5 ml base monomer + 600 μl Catalyst + 3 drops Promoter; Polysciences). Polymer was mixed and 10 min later was injected over 5 min and then left to cure on ice for 3 h. Once cured, hearts were briefly washed with ddH2O and then placed in potassium hydroxide (maceration solution) for 1 h at room temp. After maceration incubation, hearts were laid with the anterior side up and the LAD artery was imaged using a Nikon Camera (DS-12) after application of several drops of ddH2O to clear overlying tissue. Images were quantified using Imaris^®^ Filament Tracking software. First, the blue channel was extracted and changed to mono. A Gaussian filter was applied, background was subtracted, and then semi-automatic tracing was used to identify vessels and branching points. Branching points of all vessels that supply the left ventricular wall were quantified for each species. Five representative images for each species are shown in Fig. 1k, l.

### EdU administration

To assess angiogenesis, proliferating vessels were quantified in both the peri-infarct and scar regions in all strains 17 days post-MI. Mice received a single intraperitoneal injection of 5-ethynyl-2'-deoxyuridine (EdU, 10 mg/kg, Cayman Chemical, Ann Arbor, MI) immediately after MI surgery and continuing daily through post-op Day 14. Hearts were collected on Day 17 and processed as indicated in Histology and Immunofluorescence sections. Hearts were stained for Isolectin or CD31, and EdU with a DAPI counterstain. Images were taken with a ×40 oil immersion objective on a Nikon A1 Confocal Microscope in the University of Kentucky Confocal Microscopy facility. 8–15 images per section were taken and the data reported as percentage of Isolectin+ or CD31+ cells that also expressed EdU.

### Tissue collection and histology

Tissue (heart and lung) from all species were collected and weighed immediately at sacrifice. The dry lung weight was collected after 3 days of 65 °C incubation. Hearts were perfused with PBS (VWR International) followed by 4% PFA (VWR International) fixation via cannulation of the ascending aorta. Hearts were post-fixed overnight at 4 °C. Hearts were then sectioned in half long axis at the level of the ligation and transferred to 70% ethanol until sectioning. Tissues were then paraffin embedded and sectioned. All tissue was sectioned at 5 μm for immunofluorescence and 8 μm for Masson’s Trichrome and Picrosirius Red staining. Masson’s Trichrome was imaged using an Olympus BX53 microscope (Olympus, Tokyo, Japan) at ×4 and ×40 to evaluate scar. The scar size was quantified by examining and calculating the average percent of fibrosis of the total LV area using NIH ImageJ 1.46R software based on Masson’s trichrome staining. Picrosirius red staining was performed to visualize scar collagen organization at the peri-infarct and infarct regions. Images of picrosirius stained slides were captured at ×40 magnification using Olympus BX53 microscope (Olympus, Tokyo, Japan). The minimum and maximum scar cross sectional length was measured using ImageJ to determine the thickness of the scar. DAPI counterstain on ventricular heart sections was performed to analyze cellular density of scar tissue. Images of DAPI stained tissue were taken at ×4 and ×60 magnification using Olympus BX53 microscope in the University of Kentucky Light Microscopy Core. The number of nuclei in each imaging field was counted using ImageJ. All measurements were analyzed by blinded observers.

### Immunofluorescence

Immunofluorescence assessments were carried out on deparaffinized and rehydrated sections as previously described^50^. After deparaffinization, slides were washed, and TRIS (Citrate for cardiac troponin T) heat induced epitope retrieval was performed for 25 min. For Isolectin (IB4)/EdU and CD31/EdU staining, the following EdU staining was performed between the antigen retrieval and protein blocking steps. A reaction cocktail was created using 5 μl 2 M Tris (pH8.5; Thermo), 2 μl 50mMCuSO_4_ (Fisher), 20 μl 0.5 M fresh ascorbic acid (Thermo), 2 μl Alexa Fluor Azide (0.25 mg/ml, Thermo) and 73 μl ddH20 per slide. Slides were incubated in the dark in reaction cocktail for 30 min at room temperature, then washed in PBS. Protein blocking (3%) was performed for 30 min and slides were washed in PBS. Permeabilization was attained with 0.3% Triton X-100 for alpha smooth muscle actin, cardiac troponin T (cTNT) staining. IB4 stained slides were also blocked for streptavidin/biotin (SP-2002 Vector Labs) according to manufacturer’s protocol. After blocking, slides were washed in PBS and incubated with primary antibody: Isolectin B4 (L-2140, Sigma, 1:500), CD31 (AF3628, R&D Systems, 1:100), cTNT (MA5-12960, Invitrogen, 1:100) or alpha smooth muscle actin (α-SMA, MAB1420, R&D Systems, 1:50) overnight at 4 °C. After washing in PBS, sections were incubated with secondary antibody: streptavidin-Alexa Fluor 568 (S11226, Thermo Fisher,1:500) for IB4, Donkey anti goat Alexa Fluor 568 (ab175704, Abcam, 1:500) for CD31, or Donkey anti-mouse Alexa Fluor 568 (ab175700, Abcam, 1:500) for cTNT and α-SMA for 30 min at room temperature. Slides were washed and cover slipped with antifade mounting medium containing DAPI counterstain (H-1200, Vector Labs). 8–15 adjacent areas per section were examined at ×40 magnification using Nikon A1 Confocal Microscope in the University of Kentucky Confocal Microscopy facility. IB4 staining was performed on tissues from day 50 post-MI and was quantified at both the peri-infarct border and the center of the scar and is presented as total capillary density per mm^2^. Baseline IB4 measurements were taken at a comparable location to the peri-infarct area. To identify vessels in the peri-infarct and infarct regions 50 days post MI, CD31 staining was performed and the number of complete vessels with multiple nuclei and open lumen were quantified in 9–15 images per section per area in all species. To determine vessel proliferation at 17 days post-MI using either IB4/EdU or CD31/EdU, 8–15 images per section were taken and the data reported as percentage of IB4+ or CD31+ cells that also expressed EdU.

To identify extent of damage at 3 days post-MI, we stained for cTNT and Wheat Germ Agglutinin 488 (WGA488, L4895, Sigma, 1:50). In this case, WGA488 was incubated after blocking and before permeabilization steps listed above for 15 min at room temperature followed by 3 PBS washes. Images were taken on Zeiss Axioscanner for whole slice images, insets were obtained on Nikon AR1 confocal with a ×40 oil immersion lens, both located in the University of Kentucky microscope core.

### Cardiomyocyte isolation

Ventricular CMs were isolated as previously described. Briefly, animals received an i.p. injection of heparin (0.3 ml of 1000 units/ml) prior to sacrifice. Animals were then anesthetized with 1–3% Isoflurane. Hearts were excised and immediately perfused on a Langendorff apparatus with a high-potassium Tyrode buffer and then digested with 5–7 mg of liberase (Roche Applied Science). After digestion, atria were removed, and ventricular myocytes were mechanically dispersed. Some isolated ventricular CMs were used for measuring cell surface area and nuclei, and the others were used for electrophysiological recordings and calcium transients. For electrophysiology and calcium transient measurements control *Mus* were used containing Rad^fl/fl51^. Some of this control *Mus* data appeared in Ahern et al.^51^. Calcium concentrations were gradually restored to physiological levels in a stepwise fashion for electrophysiological studies, and only healthy quiescent ventricular myocytes were used for electrophysiological analysis within 12 h.

### Electrophysiological recordings and calcium transients

I_Ca,L_ was recorded in the whole-cell configuration of the patch clamp technique as previously described^51^. All recordings were performed at room temperature (20–22 °C). The pipette solution consisted of (in mmol/liter) 125 Cs-methanosulfonate, 15 TEA-Cl, 1 MgCl2, 10 EGTA, and 5 Hepes, 5 MgATP, 5 phosphocreatine, pH 7.2. Bath solution contained (in mmol/liter) 140 NaCl, 5.4 KCl, 1.2 KH2PO4, 5 Hepes, 5.55 glucose, 1 MgCl2, 1.8 CaCl2, pH 7.4. Once a cell was successfully patched, zero sodium bath solution was introduced into the chamber (mmol/liter) 150 N-methyl-D-glucamine, 2.5 CaCl2, 1 MgCl2, 10 glucose, 10 Hepes, 4-amino-pyridine, pH 7.2. Recordings of ISO response were recorded in zero sodium bath solution containing 300 nM ISO. I_Ca,L_ was recorded from a holding potential (V_hold_) of −50 mV. I_Ca,T_ and I_Ca,L_ was recorded from V_hold_ −80 mV with 300 ms depolarization steps to levels as shown in Fig. 3. Activation voltage dependence parameters were obtained by first transforming the peak current-voltage relationship to a conductance transform by fitting the ascending phase (typically V test +15 to +40 mV) to a linear regression to obtain a reversal potential. Using G = I/V the conductance as a function of voltage transform was then fitted to a Boltzmann distribution of the form G(V) = Gmax/[1+exp(V_1/2_/*k*)] where Gmax is the maximal conductance and V_1/2_ is the activation midpoint and *k* is the slope factor. For the steady state availability curve, we pre-pulsed cells to Vpre ranging from −40 to +10 mV in 5 mV increments and recorded a V_test_ to 0 mV. Peak currents were plotted as a function of Vpre, and a Boltzmann distribution was fitted to the resulting curve.

Calcium transients were recorded from ventricular CMs loaded with cell permeable Fura-2-AM (Invitrogen). CMs were field stimulated at 1.0 Hz to determine transient amplitude, upstroke velocity, and rate of decay. All measurements were made following >2 min of conditioning of 1 Hz-field stimuli to induce steady state. Transients were recorded at 1 Hz. All Ca^2+^ transient/sarcomere dynamic data were analyzed using IonOptix IonWizard 6.3 (IonOptics Inc., Westwood, MA). Background fluorescence (Fbackground) for F380 and F340 were determined from cell-free regions. Data are expressed as F340/380 and were corrected for Fbackground.

### T-tubule quantification

For T-tubule quantification we used dispersed CMs that were not re-exposed to physiological calcium to preserve resting CM length. CMs were incubated in Di-8-ANEPPS (5 mM; ThermoFisher, cat#D3167) for 5 min, cells were rinsed in low-calcium relaxation buffer, and photographed on a Zeiss5Live laser scanning confocal microscope with a ×100 oil immersion objective. Di-8-ANEPPS was excited at 488 nm and emission wavelengths >505 nm were collected for an optical slice at a z-plane level without visible nuclei. Photomicrographs were analyzed for T-tubule organization using the AutoTT program designed by Drs. Guo and Song^52^. AutoTT allows simultaneous measurement of the longitudinally oriented and transversely oriented T-tubules in each of the ANEPPS-labeled CMs. To visualize z-discs, isolated ventricular CMs were fixed in 4% PFA for 10 min at 37 °C, and washed with PBS, permeabilized, and centrifuged at 300 RPM for 3 min. Collected cells were stained with mouse anti a-actinin (Sigma A7811, lot 111M4845, 1:500 dilution), and visualized with Alexa Flour 594 donkey anti-mouse IgG (ThermoFisher, cat. A21203, lot 1722995, 1:500 dilution). Confocal Z-series stacks (step size 210 nm) were acquired on a Zeiss confocal laser scanning microscope (LSM) 880 using the Airyscan detector, a Plan-Apochromat ×63/1.4 DIC oil immersion objective. The laser wavelength and strength was 594 nm at 0.7% with a pinhole size of 2.59 AiryUnits (AU). The Zeiss Zen (black edition) software was used to detect Alexa Flour 594 in the Airyscan super resolution mode using main dichroic beam splitters 488/549 and 405. Raw.czi files were processed into deconvolved images using the Zen software. 3D reconstruction images were visualized and captured as.tif files in Zen blue version 3.4.89. Movies are a 50-frame series of images, 360-degree panorama.

### Cardiomyocyte surface area and nucleation measurement

Isolated ventricular CMs were fixed in 4% PFA for 10 min at 37 °C and washed once with PBS by centrifugation at 300 RPM for 3 min. Collected cells were stained with DAPI at 1:10,000 dilution for 5 min at 37 °C (H3570, Thermo Fisher Scientific, Waltham, MA) following by another PBS wash prior to mounting on glass slides with ProLong™ Gold Antifade mounting solution (Thermo Fisher, P36934). For surface area quantification, isolated CMs were examined using an Olympus IX-71 with DP72 color camera (12.8 megapixel cooled digital color camera). Measurement of CM size was performed using Olympus cellSens software area measuring function to trace the surface of CMs. For nucleation analysis, the number of DAPI stained nuclei/CM from different fields were quantified.

### Ploidy and nuclear volume measurement

CM ploidy analyses were done as previously described with modifications^30^. PFA fixed ventricular CMs from each group were used in ploidy and nuclear volume measurement. Confocal z-stacks of randomly selected CMs were captured using Nikon Ti, A1 confocal microscope (Nikon, Japan) with a step size of 3 μm. ×40 oil immersion objective was utilized for all acquisitions. Ploidy and nuclear volume were measured in z-stacks using Imaris Version 9 software (Bitplane AG, Switzerland). 3D Nuclei were identified and outlined with a standard threshold requirement for all groups using the Imaris cell analysis function to determine nuclear volume and mean DAPI intensity for ploidy analysis. Mean DAPI intensity of spleen cell nuclei within species were used as reference for diploid (2N) nuclei. CM nuclei were determined as diploid if their normalized intensity values were within 1–1.5 times range of reference cells. Nuclei were determined as tetraploid if their intensity values were within 1.5–2.5 times range of the reference cells. Polyploidy was assigned if DAPI intensity was greater than 2.5 times range of reference cells, as previously described^53^.

### Murine model of myocardial infarction

MI surgery was performed as previously described^50^. In brief, we anesthetized mice with 1–3% isoflurane using a small animal vaporizer system. The pain reflex was examined to make sure that the mice were adequately anaesthetized before surgery. A thoracotomy was performed between the 4th and 5th ribs and the pericardial sac was removed. The heart was exposed and pushed out of the chest. The LAD coronary artery was identified under direct vision and was permanently ligated 3 mm below its origin using 6–0 silk suture. After LAD ligation, the heart was returned to the intrathoracic space, the muscles were closed, and the skin was sutured using 4–0 prolene running sutures.

### Echocardiography

A heating pad and rectal temperature probe were utilized to keep body temperature at 37 °C during the experiment. Modified parasternal long-axis and short-axis was utilized to determine left ventricular function and volume in M-mode, two-dimensional and Doppler echocardiography modes. We also used M-mode tracings at the mid-papillary level to estimate the systolic and diastolic parameters, and Teichholz formula at end-systole and end-diastole to measure the left ventricle (LV) volumes. All animals were anaesthetized using 1–3% isoflurane during Echocardiography to maintain a heart rate of 450–500 BPM for all echocardiographic acquisitions. Echo was performed at baseline and at D2, D7, D14, D30, and D50 post-MI.

### Cardiac magnetic resonance imaging

Cardiac magnetic resonance imaging (CMR) was performed on a 7-Tesla ClinScan system (Bruker, Ettlingen, Germany, http://www.bruker.com) equipped with a 4-element phased-array cardiac coil and a gradient system with a maximum strength of 450 mT/m and a maximum slew rate of 4500 mT/m/s. We acquired whole short-axis stack images from base to apex for comparison with late enhancement images. The short-axis images were planned perpendicular to the four-chamber long-axis image. For late gadolinium-enhanced magnetic resonance imaging, a 0.6 mmol/kg bolus of gadolinium-diethylenetriamine pentaacetic acid (Gd-DTPA; Gadavist, Bayer Health Care, Whippany, NJ) was injected using the intraperitoneal route. Imaging was initiated 10 min after the injection of Gd-DTPA using an electrocardiographically gated segmented magnetization-prepared fast low-angle shot sequence with a fixed inversion time at 500 ms. CMR was performed at D3 and D14 post-MI. The CMR data were analyzed using commercially available post-processing software: CMR42 (Circle Cardiovascular Imaging, Calgary, Alberta, Canada, http://www.circlecvi.com) and ImageJ (NIH). Infarct length was calculated as a percentage of left ventricular length. All analyses were performed by blinded investigators.

### Statistics

Values are expressed as mean ± standard error of mean (s.e.m). We used student *T*-test, one-way-ANOVA, repeated-measures ANOVA and mixed-effects ANOVA with Tukey’s corrections to compare data across species as appropriate. The mixed-effects model integrates analysis that uses the available data to estimate model parameters. Animals with partial data contribute to the estimation of some model parameters but not others. It is important to note that relying solely on repeated-measures ANOVA will limit the sample size to only animals that survived for the entire duration of the study and could lead to selection bias as we are only selecting a subgroup of animals. Throughout the manuscript, analyses that compared species and time but did not include paired samples such as data in Fig. 4g, h were analyzed using 2-way independent ANOVA. Sample sizes, statistical tests and *P* values are indicated in the figures or figure legends. Animal numbers are presented as a range based on the number of animals included in each analysis. These numbers are included in the figure legends. Throughout the analyses, a *P* value < 0.05 was considered statistically significant.

### Reporting summary

Further information on research design is available in the Nature Research Reporting Summary linked to this article.

## Supplementary information

Supplementary Movie 1

Supplementary Movie 2

Supplementary Movie 3

Supplementary Movie 4

Supplementary Movie 5

Supplementary Information

Reporting Summary

## Data Availability

The authors declare that all the data supporting the findings of this study are available within the paper and are contained within Supplementary Information.
