# Author Correction: Adult spiny mice (*Acomys*) exhibit endogenous cardiac recovery in response to myocardial infarction

**DOI:** 10.1038/s41536-023-00314-2

**Published:** 2023-07-15

**Authors:** Hsuan Peng, Kazuhiro Shindo, Renée R. Donahue, Erhe Gao, Brooke M. Ahern, Bryana M. Levitan, Himi Tripathi, David Powell, Ahmed Noor, Garrett A. Elmore, Jonathan Satin, Ashley W. Seifert, Ahmed Abdel-Latif

**Affiliations:** 1 Saha Cardiovascular Research Center, College of Medicine, University of Kentucky, Lexington, KY USA; 2 The Center for Translational Medicine, Lewis Katz School of Medicine, Temple University, Philadelphia, PA USA; 3 Department of Physiology, College of Medicine, University of Kentucky, Lexington, KY USA; 4 Gill Heart and Vascular Institute and Division of Cardiovascular Medicine, University of Kentucky, Lexington, KY USA; 5 Department of Biology, University of Kentucky, Lexington, KY USA; 6 The Lexington VA Medical Center, Lexington, KY USA; 7 Division of Cardiovascular Medicine, Department of Internal Medicine, University of Michigan, Ann Arbor, MI USA

**Keywords:** Cardiac regeneration, Experimental models of disease

Correction to: *npj Regenerative Medicine* 10.1038/s41536-021-00186-4, published online 17 November 2021

In this article panels a and b of Figure 7 were accidentally interchanged with panels a and b of Supplementary Figure 6. The original article has been corrected.
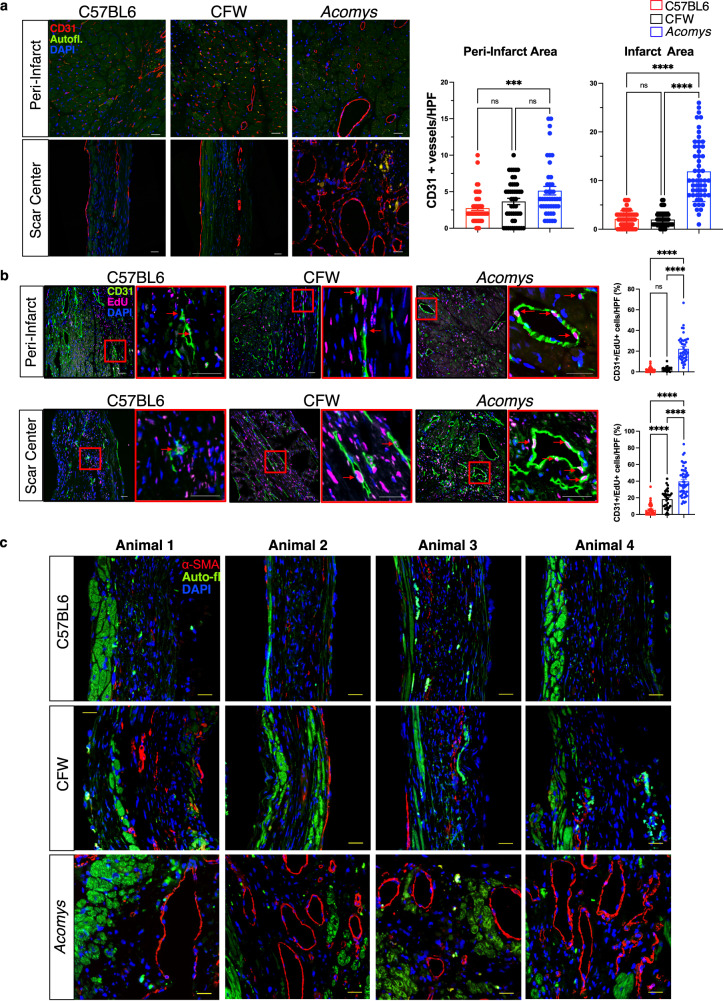


## Supplementary information

Supplementary information

