# Supplementary material for: Adult spiny mice (Acomys) exhibit endogenous cardiac recovery in response to myocardial infarction
Source: NPJ Regen Med. 2021 Nov 17;6:74. doi: 10.1038/s41536-021-00186-4 (PMC8599698; doi:10.1038/s41536-021-00186-4)
Supplement: Supplementary file 6 — Supplementary Information [file 41536_2021_186_MOESM6_ESM.pdf]

Supplementary Figures

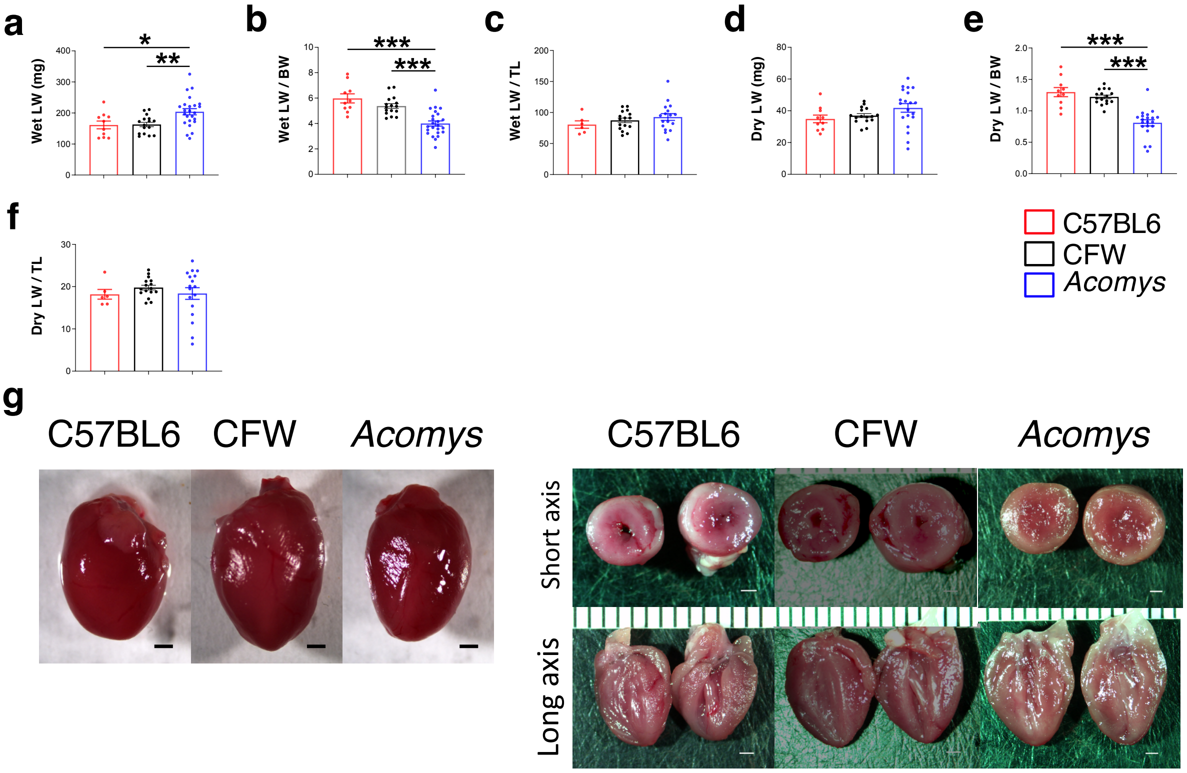

Supplementary Figure 1. Cardiac function, gravimetric and vascular characterization across mouse strains. (a-f) Quantitation of dry and wet lung weight of *Mus*-C57BL6 (N = 10), *Mus*-CFW (N = 19) and *Acomys* (N = 22). Analyses demonstrate comparable lung weight across species when normalized by body weight (BW) and tibia length (TL) (values are means ± S.E.M, **P* < 0.05, ***P*<0.01, and ****P*<0.001 by one-way ANOVA and Dunnett's correction with Acomys as control). (g) Representative whole hearts, short axis and long axis *Mus* (C57BL6) (left), *Mus* (CFWß) (middle) and *Acomys* (right) at baseline.

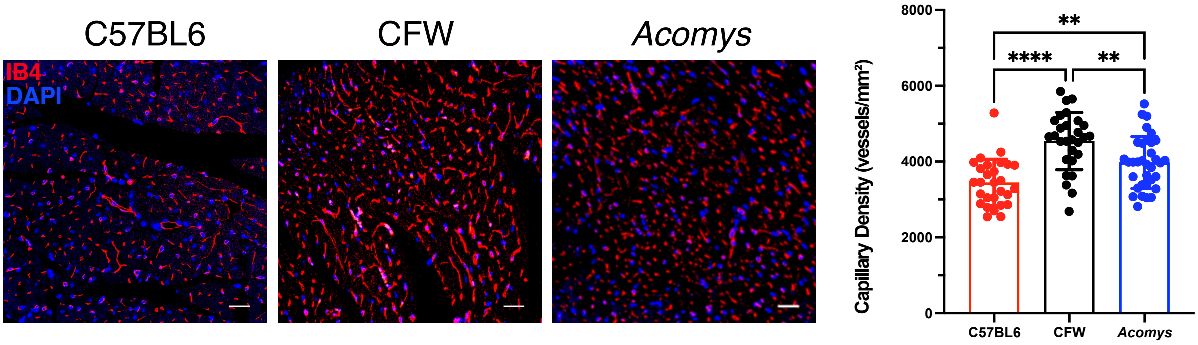

Supplementary Figure 2. Baseline capillary density across species. Representative image of isolectin B4 staining of cardiac tissue at baseline in all species. Quantitative analysis showed higher capillary density in CFW-*Mus* compared to C57BL6-*Mus* and *Acomys* (N= 3 animal/group, values are means ± S.E.M, ***P*< 0.01 by one-way ANOVA and Tukey's multiple comparison test).

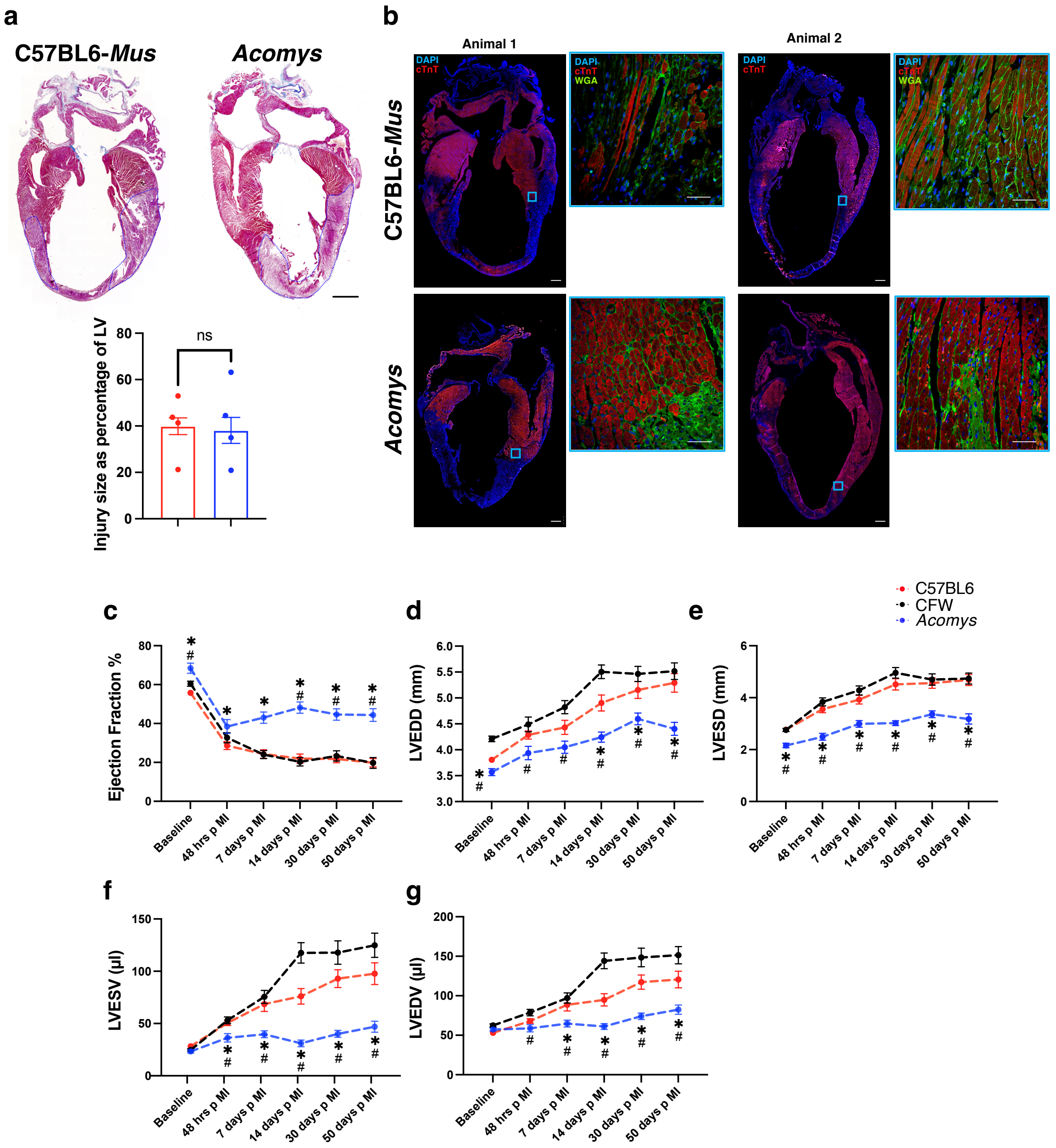
a
C57BL6-Mus
Acomys
ns
Injury size as percentage of LV
0
20
40
60
80
b
Animal 1
Animal 2
C57BL6-Mus
Acomys
DAPI
cTnT
WGA
c
d
e
f
g
C57BL6
CFW
Acomys
Ejection Fraction %
LVEDD (mm)
LVESD (mm)
LVESV (µl)
LVEDV (µl)
Baseline
48 hrs p MI
7 days p MI
14 days p MI
30 days p MI
50 days p MI

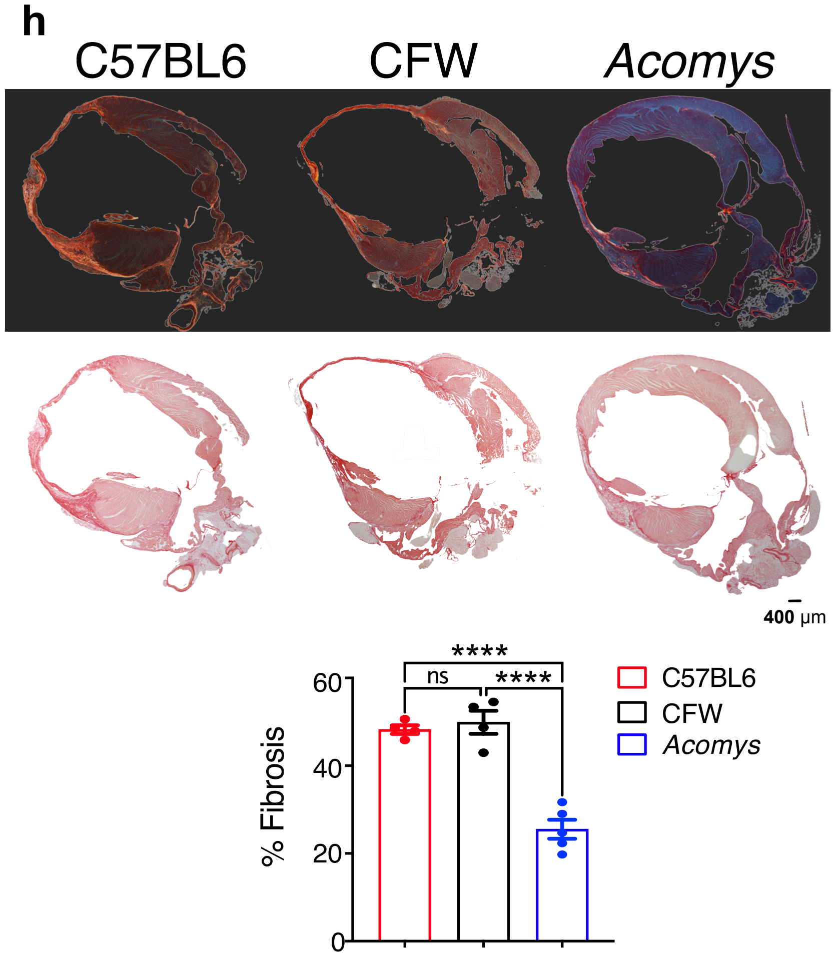

Supplementary Figure 3. Lower scar progression and functional stabilization observed in *Acomys* but not *Mus* after MI. (a) Representative images of Masson's trichrome stained long-axis left ventricular cavity sections of *Mus* (C57BL6) and *Acomys* at 3 days post-MI (scale bar = 1 mm). Quantification of the size of myocardial injury, showing initial injury in *Acomys* and *Mus* strains (N= 4 C57BL6-*Mus* and 4 *Acomys, P*=0.79 using *t*-test). (b) Representative images of cardiac troponin T (cTnT) and wheat germ agglutinin (WGA) stained long-axis left ventricular cavity sections of *Mus* (C57BL6) and *Acomys* at 3 days post-MI depicting the range of damage from least to most severe (lower panel, scale bar = 500 μm) indicating full range of damage at D3. Insets show the morphology of cardiomyocytes at the border of infarct (scale bar = 50 μm). Echocardiographic recordings of left ventricular ejection fraction (c), left ventricular end-diastolic diameter (LVEDD) (d), left ventricular end-systolic diameter (LVESD) (e), left ventricular end-systolic volume (LVESV) (f), and left ventricular end-diastolic volume (LVEDV) (g) at baseline and 2, 7, 14, 30, 50 days after MI (N = 16-45 *Mus*-C57BL6, 10-20 *Mus*-CFW and 8-11 *Acomys*). These data suggest similar injury pattern early after MI across species with preservation of cardiac

function in *Acomys* but not *Mus* strains in long-term functional follow-up (values are means ± S.E.M, $^{*\#}P < 0.05$ by Mixed-effects ANOVA, compared to **Mus*-C57BL6 or $^{\#}$*Mus*-CFW).

(h) Representative images of Picrosirius red stained mid left ventricular cavity sections of *Mus* (C57BL6 and CFW) and *Acomys* 50 days post-MI (scale bar = 400 μm). Quantification of fibrosis, corresponding to the ratio between infarcted length and left ventricular length showing significantly smaller fibrosis extent in *Acomys* compared to *Mus* strains (N = 4 *Mus*-C57BL6, 4 *Mus*-SWR, and 5 *Acomys*, values are means ± S.E.M, $F = 45.32$, $^{****}P < 0.0001$ by one-way ANOVA and Tukey's multiple comparison test).

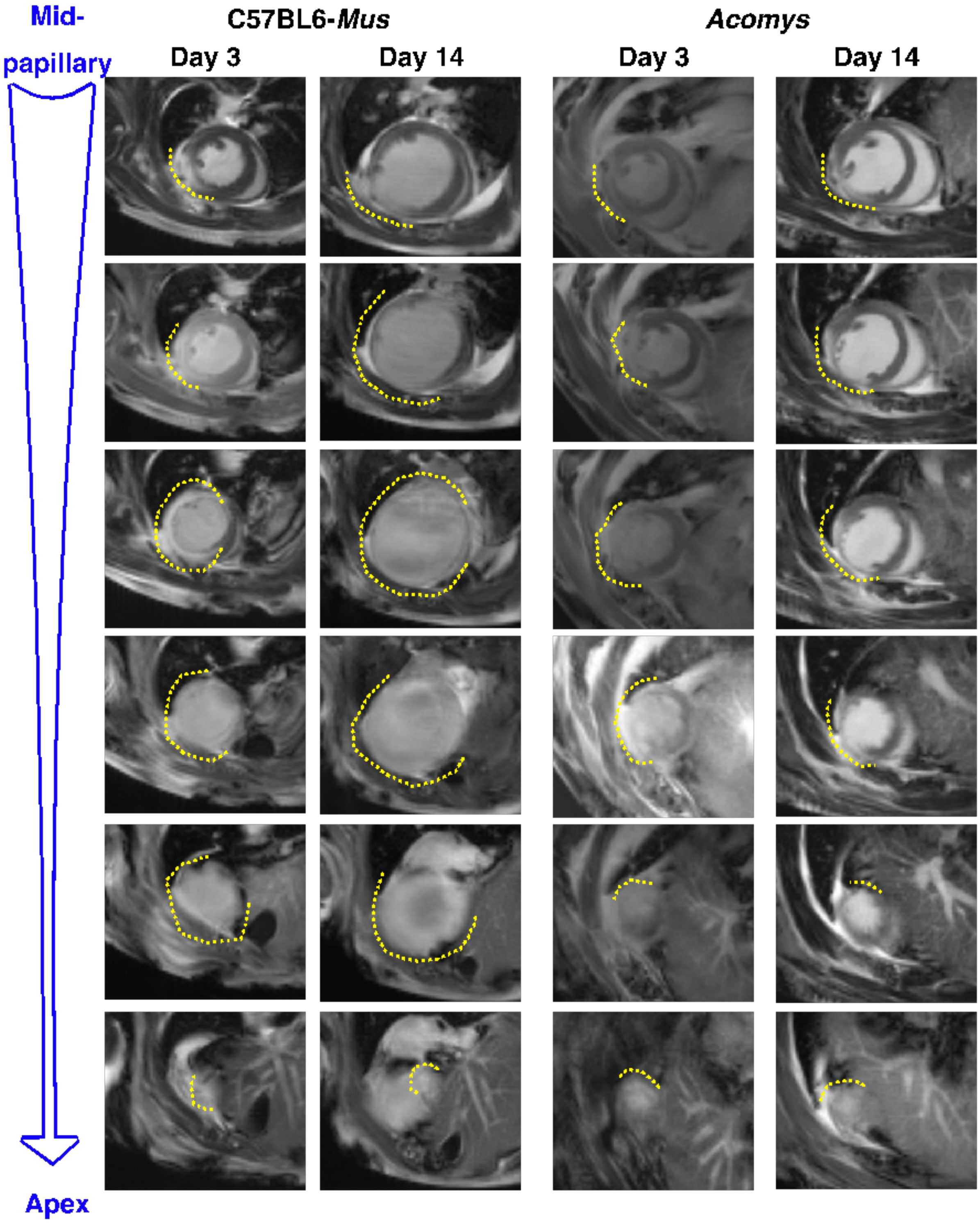

Supplementary Figure 4. **Comparable early injury and reduced subsequent infarct expansion in *Acomys* compared to *Mus*.** Serial representative CMR images from the mid papillary muscle all the way through the distal anterior wall/apex levels in a single C57BL6-*Mus* and *Acomys* showing involvement of the distal anterior wall/apex in both species at D3. While the scar is stabilized or regressed slightly in Acomys, C57BL6-*Mus* mice show evidence of infarct expansion by 14 days after injury.

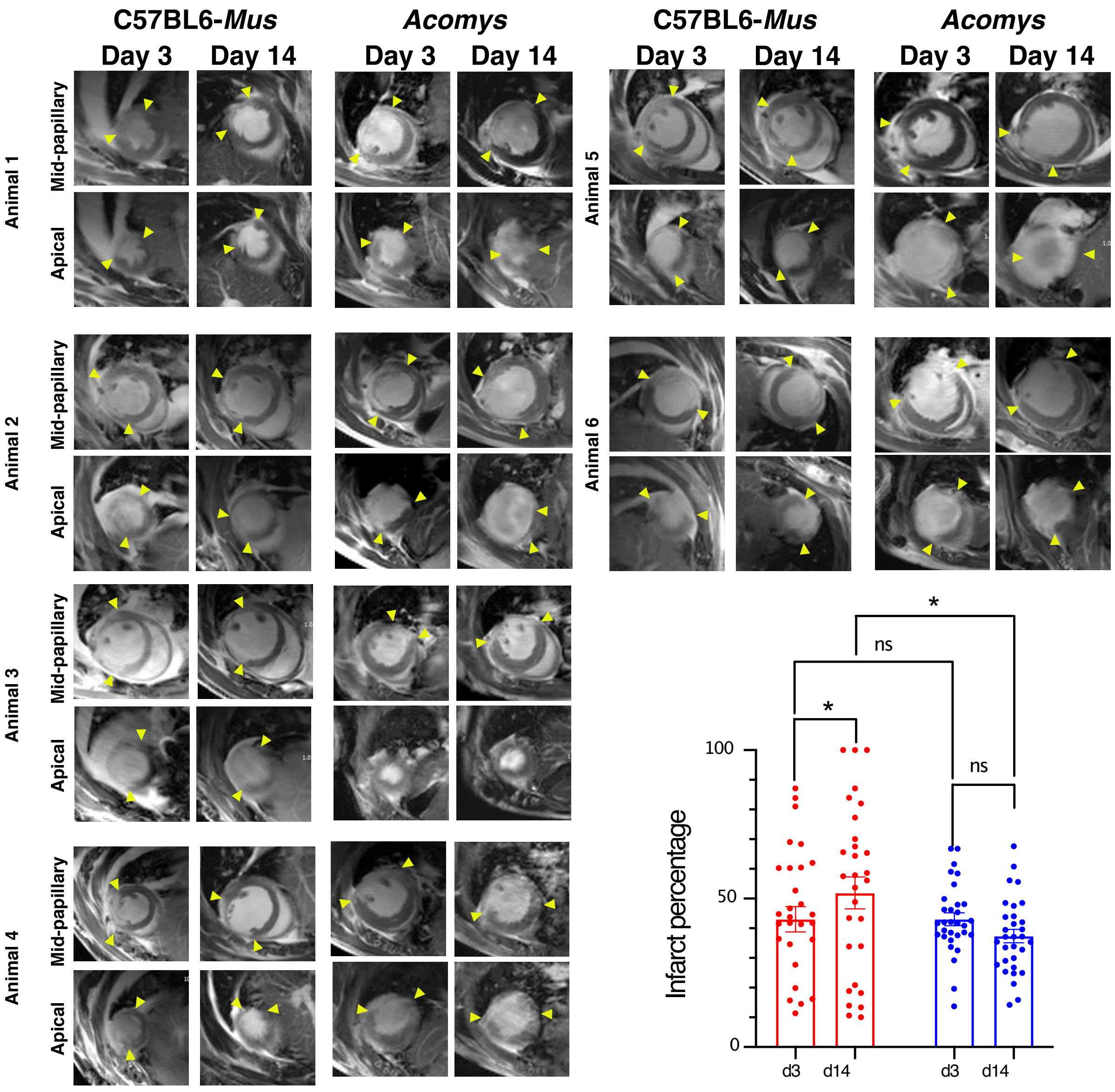

Supplementary Figure 5. **Reduced infarct expansion in *Acomys* compared to *Mus*.** Representative cardiac magnetic resonance images showing comparable medium-sized infarct area in both species at 3 days after MI. The progression of infarct area was significantly less in *Acomys* compared to *Mus* at 14 days after MI. The images show transverse sections of the left ventricle starting at the left anterior descending artery ligation site (top) and extending to the apex (bottom). Quantitative assessment of injury size as percentage of left ventricle at 3 and 14 days after MI (N = 9-10 animals per group). All values are means ± S.E.M, **P* < 0.05 compared to C57BL6-Mus by repeated measures ANOVA.

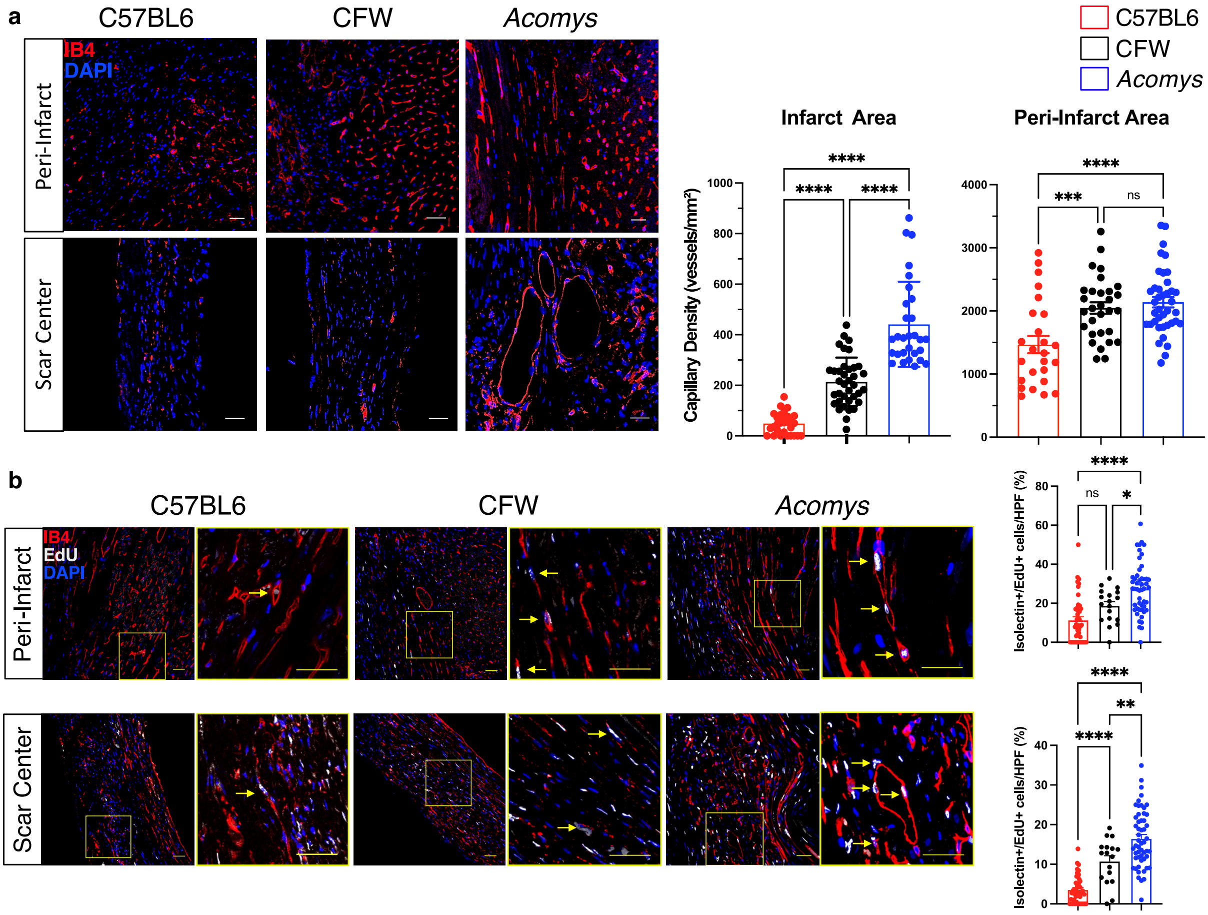

Supplementary Figure 6. Enhanced angiogenesis and vessel maturation in the infarct region following MI in *Acomys* compared to *Mus* strains. (a) Representative images of isolectin B4 staining (red) defining endothelial cells in the peri-infarct region and the center of the scar at the left ventricular cavity level of *Mus* (C57BL6 and CFW) and *Acomys* 50 days post-MI (scale bar = 25 µm). Quantification of capillary density showing significantly higher capillary density in *Acomys* compared to *Mus*-C57BL6 in the peri-infarct region and to both *Mus* strains in the center of the scar (N= 4 *Mus*-C57BL6, 4 *Mus*-CFW, and 5 *Acomys,* values are means ± S.E.M). (b) Representative images of isolectin (red) and EdU staining (white) marking proliferating endothelial cells in the peri-infarct region and the center of the scar at the left ventricular cavity level of *Mus* (C57BL6 and CFW) and *Acomys* 17 days post-MI (scale bar = 25 µm). Quantification of isolectin+/EdU+ cells showing significantly higher endothelial cell proliferation in *Acomys* compared to the two *Mus* strains in both peri-infarct and scar center areas (N= 4 *Mus*-C57BL6, 4 *Mus*-CFW, and 5 *Acomys,* values are means ± S.E.M, *$P < 0.05$, **$P < 0.01$, ***$P < 0.001$ and ****$P < 0.0001$ by one-way ANOVA and Tukey's multiple comparison test).

Supplementary Movie 1. A single *Acomys* mononuclear, ventricular cardiomyocyte immunostained with a-actinin. A z-stack of images was reconstructed and rotated to elucidate the regular ordered structure of the z-discs in these relatively small cardiomyocytes.

Supplementary Movie 2. Long axis cardiac MRI imaging of the left ventricle of C57BL6 mice 3 days after acute myocardial infarction showing the reduced myocardial contractility in the distal anterior and apical walls.

Supplementary Movie 3. Long axis cardiac MRI imaging of the left ventricle of C57BL6 mice 14 days after acute myocardial infarction showing wall thinning, adverse cardiac remodeling and ventricular cavity enlargement as well as reduced myocardial contractility in the anterior and apical walls.

Supplementary Movie 4. Long axis cardiac MRI imaging of the left ventricle of *Acomys* 3 days after acute myocardial infarction showing the reduced myocardial contractility in the distal anterior and apical walls.

Supplementary Movie 5. Long axis cardiac MRI imaging of the left ventricle of *Acomys* 14 days after acute myocardial infarction showing myocardial preservation compared to C57BL6 mice with reduced adverse remodeling, less ventricular cavity enlargement and reduction of the area damage to the distal anterior and apical walls.

Supplemental Table 1: Baseline gross anatomical characteristics and cardiac function pre- and post-myocardial infarction:

		Mus (C57BL6)			*Mus* (SWR)			*Acomys*			*P-value*		
		n	Mean	SEM	n	Mean	SEM	n	Mean	SEM	*Acomys* vs *Mus* (C57BL6)	*Mus* (SWR) vs *Mus* (C57BL6)	*Acomys* vs *Mus* (SWR)
**Baseline structural parameters**													
BW (mg)	Baseline	10	26.92	1.12	19	40.28	0.66	22	50.26	2.18	<0.0001*	0.0106*	0.0019*
HW (mg)	Baseline	10	167.58	10.41	10	222.32	9.34	23	231.07	12.92	0.01*	0.0143*	>0.9999
HW/BW	Baseline	10	6.22	0.29	10	5.10	0.17	23	4.59	0.34	0.0045*	0.1116	>0.9999
HW/TL	Baseline	6	96.6	8.31	10	117.14	5.51	17	95.81	5.59	>0.9999	0.1904	0.0191*
Wet LW (mg)	Baseline	10	161.4	12.55	16	162.88	7.25	20	204.03	9.92	0.0435*	>0.9999	0.0182*
Dry LW (mg)	Baseline	10	34.91	2.42	16	36.96	1.26	20	41.83	2.71	0.0575	>0.9999	0.2989
Wet LW/BW	Baseline	10	5.98	0.36	16	5.38	0.18	20	4	0.2	0.0001*	>0.9999	0.0004*
Dry LW/BW	Baseline	10	1.3	0.07	16	1.22	0.03	20	0.81	0.05	<0.0001*	>0.9999	<0.0001*
Wet LW/TL	Baseline	6	80.62	5.99	16	87.34	3.78	17	92.72	5.45	0.5355	>0.9999	>0.9999
Dry LW/TL	Baseline	6	18.19	1.15	16	19.78	0.57	17	18.38	1.39	>0.9999	0.7238	>0.9999

**Baseline cardiac function and structural characterization**													
EF (%)	Baseline	59	55.8	1.0	35	60.5	1.3	21	68.4	2.6	0.003*	0.034	0.056
FS (%)	Baseline	24	34.10	0.77	30	35.36	1.11	10	35.01	1.35	0.7274	0.3711	0.9793
LVEDD (mm)	Baseline	58	3.8	0.04	35	4.3	0.05	21	3.6	0.1	0.014*	<0.001*	<0.001*
LVESD (mm)	Baseline	60	2.8	0.04	35	2.8	0.4	21	2.2	0.1	<0.001*	0.98	<0.001*
LVAW (mm)	Baseline	30	0.78	0.02	30	0.92	0.02	10	0.88	0.02	0.044*	<0.0001*	>0.9999
LVPW (mm)	Baseline	30	0.82	0.02	30	0.93	0.03	10	0.90	0.04	0.1909	0.002*	0.7358
LV mass/BW	Baseline	30	3.31	0.07	29	3.14	0.09	10	2.42	0.10	<0.0001*	0.6993	0.0011*
Stroke volume/BW	Baseline	30	1.61	0.03	29	1.01	0.04	10	0.98	0.05	<0.0001*	<0.0001*	>0.9999
**Cardiac recovery after MI**													
HW/BW	Day 1	3	7.402	0.3242	3	5.245	0.363	3	3.664	0.1998	<0.0001*	0.0028*	0.0226*
	Day 3	5	7.653	0.4229	2	5.989	0.268	9	3.611	0.134	0.0061*	0.4014	0.1587
	Day 7	5	8.64	0.5366	4	5.851	0.147	5	4.839	0.3321	0.0001*	0.0167*	0.4842
	Day 50	26	8.947	0.4823	12	7.9	0.495	7	4.255	0.1658	<0.0001*	0.1423	<0.0001*

Wet-Dry LW/BW	Day 1	8	5.469	0.23 11	3	4.349	0.36 3	7	3.178	0.2201	0.004*	0.43	0.412
	Day 3	6	5.253	0.24 93	5	4.53	1.15 5	6	3.752	0.2609	0.123	0.643	0.6
	Day 7	5	5.449	0320 8.	3	6.425	1.15 2	5	3.561	0.1435	0.06	0.608	0.011*
	Day 50	26	5.546	0.36 38	20	4.222	0.24 9	7	2.917	0.1288	<0.0001*	0.003*	0.071
Infarct length (%)	Day 50	17	42.67	2.21	11	44.85	2.22	5	25.37	1.94	0.0006*	0.7666	0.0003*
**Cardiac function after MI**													
EF (%)	Baseline	59	55.8	1.0	35	60.5	1.3	21	68.4	2.6	0.003*	0.034*	0.056*
	Day 2	34	28.6	1.9	14	32.7	2.6	11	38.4	3.6	0.048*	0.99	0.528
	Day 7	25	24.3	2.2	25	24.03	2.1	18	43.01	2.9	0.013*	0.99	0.53
	Day 14	32	21.95	2.3	20	20.3	2.2	19	48.2	2.9	<0.0001*	0.99	<0.0001*
	Day 30	31	21.8	1.9	19	23.3	2.6	22	44.6	2.9	<0.0001*	0.99	0.002*
	Day 50	31	19.8	2.1	17	19.7	2.8	19	44.4	3.3	<0.001*	0.6933	0.002*
LVEDD (mm)	Baseline	58	3.8	0.04	35	4.3	0.05	21	3.6	0.1	0.014*	<0.001*	<0.001*
	Day 2	34	4.3	0.08	14	4.5	0.2	11	3.9	0.1	0.08	0.44	0.02*
	Day 7	24	4.4	0.1	25	4.8	0.1	21	4.1	0.1	0.09	0.1	0.03*

	Day 14	29	4.9	0.2	20	5.5	0.1	21	4.2	0.1	0.002*	0.01	<0.001*
	Day 30	29	5.2	0.2	19	5.5	0.2	22	4.6	0.1	0.02*	0.34	<0.001*
	Day 50	29	5.3	0.2	17	5.5	0.2	19	4.4	0.1	<0.001*	0.62	<0.001*
LVESD (mm)	Baseline	60	2.8	0.04	35	2.8	0.4	21	2.2	0.1	<0.001*	0.98	<0.001*
	Day 2	34	3.6	0.1	14	3.8	0.6	11	2.5	0.1	<0.001*	0.36	<0.001*
	Day 7	26	3.9	0.2	25	4.3	0.9	20	2.99	0.1	<0.001*	0.29	<0.001*
	Day 14	30	4.5	0.2	20	4.95	0.9	21	3.02	0.09	<0.001*	0.31	<0.001*
	Day 30	30	4.6	0.2	19	4.7	0.95	22	3.4	0.1	<0.001*	0.89	<0.001*
	Day 50	30	4.7	0.2	17	4.73	0.9	18	3.2	0.2	<0.001*	0.98	<0.001*
LVESV (µL)	Baseline	48	28.2	1.3	35	24.7	1.1	35	23.3	1.6	0.054	0.12	0.77
	Day 2	34	50.5	2.5	14	53.01	3.3	17	36.3	4.01	0.015*	0.8	0.009*
	Day 7	34	68.6	6.9	25	75.5	6.2	24	39.8	3.3	0.001*	0.74	<0.001*
	Day 14	33	76.01	7.5	20	117.6	9.8	20	31.03	3.2	<0.001*	0.004*	<0.001*
	Day 30	33	92.9	8.5	19	117.9	11.2	22	40.1	3.3	<0.001*	0.19	<0.001*
	Day 50	33	97.7	10.4	17	124.8	11.6	19	46.97	5.3	<0.001*	0.20	<0.001*
LVEDV (µL)	Baseline	45	53.01	1.9	35	62.4	2.02	35	57.2	2.02	0.29	0.004*	0.18
	Day 2	34	67.99	2.9	14	78.7	4.03	17	58.8	4.5	0.22	0.09	0.008*
	Day 7	32	88.5	7.71	25	97.1	6.42	24	64.8	4.4	0.03*	0.67	0.004*
	Day 14	31	94.8	7.8	20	144.1	9.9	20	61.1	3.7	0.001*	0.001*	<0.001*
	Day 30	31	117.2	8.95	19	148.5	11.8	22	74.03	4.1	<0.001*	0.1	<0.001*

Day 50	31	120.6	10.5	17	151.3	10.9	19	82.4	5.8	0.007*	0.12	<0.001*

BW; body weight, HW; heart weight, TL; tibia length, LW; lung weight, EF; ejection fraction, LVEDD; left ventricular end-diastolic diameter, LVESD; left ventricular end-systolic diameter, LVAW; left ventricular anterior wall, LVPW; left ventricular posterior wall, LV mass; left ventricular mass (*$P < 0.05$).
