# Supplementary material for: Adult spiny mice (Acomys) exhibit endogenous cardiac recovery in response to myocardial infarction
Source: NPJ Regen Med. 2021 Nov 17;6:74. doi: 10.1038/s41536-021-00186-4 (PMC8599698; doi:10.1038/s41536-021-00186-4)
Supplement: Supplementary file 7 — Reporting Summary [file 41536_2021_186_MOESM7_ESM.pdf]

nature research

Corresponding author(s): Ashley W. Seifert, Ahmed Abdel-Latif, Jonathan Satin

Last updated by author(s): Jun 1, 2021

Reporting Summary

Nature Research wishes to improve the reproducibility of the work that we publish. This form provides structure for consistency and transparency in reporting. For further information on Nature Research policies, see our Editorial Policies and the Editorial Policy Checklist.

Statistics

For all statistical analyses, confirm that the following items are present in the figure legend, table legend, main text, or Methods section.

n/a	Confirmed	
☐	☒	The exact sample size (*n*) for each experimental group/condition, given as a discrete number and unit of measurement
☐	☒	A statement on whether measurements were taken from distinct samples or whether the same sample was measured repeatedly
☐	☒	The statistical test(s) used AND whether they are one- or two-sided<br>*Only common tests should be described solely by name; describe more complex techniques in the Methods section.*
☒	☐	A description of all covariates tested
☒	☐	A description of any assumptions or corrections, such as tests of normality and adjustment for multiple comparisons
☐	☒	A full description of the statistical parameters including central tendency (e.g. means) or other basic estimates (e.g. regression coefficient) AND variation (e.g. standard deviation) or associated estimates of uncertainty (e.g. confidence intervals)
☐	☒	For null hypothesis testing, the test statistic (e.g. *F*, *t*, *r*) with confidence intervals, effect sizes, degrees of freedom and *P* value noted<br>*Give P values as exact values whenever suitable.*
☒	☐	For Bayesian analysis, information on the choice of priors and Markov chain Monte Carlo settings
☒	☐	For hierarchical and complex designs, identification of the appropriate level for tests and full reporting of outcomes
☒	☐	Estimates of effect sizes (e.g. Cohen's *d*, Pearson's *r*), indicating how they were calculated

Our web collection on statistics for biologists contains articles on many of the points above.

Software and code

Policy information about availability of computer code

Data collection	No software was used
Data analysis	Provided in relevant section of methods

For manuscripts utilizing custom algorithms or software that are central to the research but not yet described in published literature, software must be made available to editors and reviewers. We strongly encourage code deposition in a community repository (e.g. GitHub). See the Nature Research guidelines for submitting code & software for further information.

Data

Policy information about availability of data

All manuscripts must include a data availability statement. This statement should provide the following information, where applicable:

- Accession codes, unique identifiers, or web links for publicly available datasets
- A list of figures that have associated raw data
- A description of any restrictions on data availability

The authors declare that all the data supporting the findings of this study are available within the paper and are contained within supplementary information.

Field-specific reporting

Please select the one below that is the best fit for your research. If you are not sure, read the appropriate sections before making your selection.

☒ Life sciences ☐ Behavioural & social sciences ☐ Ecological, evolutionary & environmental sciences

For a reference copy of the document with all sections, see nature.com/documents/nr-reporting-summary-flat.pdf

Life sciences study design

All studies must disclose on these points even when the disclosure is negative.

Sample size

We used 80% power at a two-sided 5% significance level and hence the sample size per group is approximately 16/E2, where E denotes the effect size: difference in group means, divided by group standard deviation. A sample size of 36 AMI and 24 sham mice per group in an experiment, allowing for ~20% attrition, will provide 5-6 mice per group at each time-point and allowed us to examine both short- and long-term endpoints.

Data exclusions

Exclusion of animals was set a priori and was done for animals that did not have MI on the follow up echocardiography (48 hours and 7 days), I.e. animals that did not have drop in cardiac function on echocardiography. Although we rarely encountered this, when we did, these animals were excluded from all analyses.

Replication

The MI study was replicated at least twice for spiny mice and the SWR strains. It was replicated three times for B6. The results were reproducible for myocardial preservation and smaller scar size. Each heart dispersal is a replication. For single cell measurements we reported N=mice and n=cells. For CM parameters, only intact cells were used. Broken cardiomyocytes were excluded from size, nucleation and nuclear analysis.

Randomization

Cages of up to five animals per strain were randomly assigned to group. This information is included in the text in the Methods section, under "Statistics and Grouping" in the last paragraph of Page 6

Blinding

Due to the different appearance of animals, the surgeon was not blinded to the groups. However, all analyses including echocardiography, flow cytometry and immunohistochemistry were performed blindly.

Reporting for specific materials, systems and methods

We require information from authors about some types of materials, experimental systems and methods used in many studies. Here, indicate whether each material, system or method listed is relevant to your study. If you are not sure if a list item applies to your research, read the appropriate section before selecting a response.

Materials & experimental systems

n/a	Involved in the study	
☐	☒	Antibodies
☒	☐	Eukaryotic cell lines
☒	☐	Palaeontology and archaeology
☐	☒	Animals and other organisms
☒	☐	Human research participants
☒	☐	Clinical data
☒	☐	Dual use research of concern

Methods

n/a	Involved in the study	
☒	☐	ChIP-seq
☒	☐	Flow cytometry
☒	☐	MRI-based neuroimaging

Antibodies

Antibodies used

Primary antibodies used: Isolectin B4 (L-2140, Sigma), alpha smooth muscle actin (MAB1420, R&D Systems). Secondary antibodies: streptavidin-Alexa Fluor 568 (S11226, Thermo Fisher), anti mouse Alexa Fluor 568 (ab175700, Abcam)

Validation

Primary antibodies- two primary antibodies were used in this study.
Isolectin GS-IB4 (Sigma #L-2140). Sigma provides citations on their website for the use of isolectin to associate with endothelial cells. For each mouse strain tested and for spiny mice, we ran a negative (no primary antibody) control. 8-20 images per animal were quantified. Representative images and graphs of the quantification are located in Figure 7A and 7B.

Alpha Smooth Muscle Actin (R & D system MAB1420). R&D Systems provides citations on their website for the use of this antibody to associate with alpha-smooth muscle actin. The antibody has broad specificity across human, mouse and rat. Vendor validation with Western Blot, flow cytometry and Dual RNAscope ISH/IHC. For each mouse strain tested and for spiny mice, we ran a negative (no primary antibody) control. We took images from 4 animals per group, and these are represented in Figure7C.

Animals and other organisms

Policy information about studies involving animals; ARRIVE guidelines recommended for reporting animal research

Laboratory animals	Male Acomys cahirinus (6-8 months old, sexually mature animals) were obtained from our in-house breeding colony. Male Mus musculus (8-12 weeks, sexually mature animals) C57BL6 were obtained from the Jackson Laboratory, Bar Harbor, ME and outbred SWR were obtained from Charles River.
Wild animals	The study did not use wild animals.
Field-collected samples	The study did not use field collected samples.
Ethics oversight	All animal procedures were approved by the University of Kentucky Institutional Animal Care and Use Committee (IACUC #: 2019-3254, 2013-1155 and 2011-0889).

Note that full information on the approval of the study protocol must also be provided in the manuscript.
